# Leader-Selection Mechanisms and Matching Protocols in Public Goods Games: Evidence from the Laboratory

**DOI:** 10.3390/bs16081333

**Published:** 2026-08-03

**Authors:** Dongsheng Chen, Jing Liu, Wenhao Zhang, Jie Zheng

**Affiliations:** 1 Business School, Xiangtan University, Xiangtan 411100, China; dschen@xtu.edu.cn (D.C.);; 2 Center for Economic Research, Shandong University, Jinan 250100, China; wenhaozhang2024@mail.sdu.edu.cn

**Keywords:** public goods, cooperation, leadership, controlled online laboratory experiment, signaling

## Abstract

Using a controlled online laboratory experiment, we investigate how leader-selection rules influence public goods provision under fixed matching and random rematching. In our design, a leader is a first mover who chooses a contribution before the other group members, and this contribution is revealed to the later mover(s) before they make their own decisions. In leaderless groups, all group members choose their contributions simultaneously, so no member observes another member’s contribution before deciding. Leadership does not produce a uniform increase in contributions. Among the mechanisms examined, delegated leadership by a randomly selected candidate (DL-RC) exhibited the lowest overall mean contribution, particularly under random rematching; however, the primary analyses did not establish a statistically significant general mechanism effect. Within VL-RC, the accepted one-leader branch exhibited higher observed contributions than the refusal-contingent leaderless branch; within DL-RC, the accepted one-leader branch exhibited higher observed contributions than the refusal-contingent three-first-mover branch. Because these realized branches are generated by candidates’ acceptance decisions, the comparisons are conditional and do not identify causal effects of acceptance, refusal, leader absence, or the number of first movers. Across leader-present states, later-mover contributions were positively associated with the observed mean contribution of the first mover(s). Fixed matching yielded a higher observed mean contribution than random rematching, but the pooled difference was not statistically significant; the clearest differences in matching protocols arose in DL-RC.

## 1. Introduction

Mitigating free-riding in the provision of public goods fundamentally relies on addressing coordination failures and incentivizing voluntary contributions. A growing body of the literature highlights “leading by example” as an important mechanism to promote cooperation (Glowacki & von Rueden, 2015; Pietraszewski, 2020). In the present study, we use the term leader in a specific experimental sense: a leader is the participant who makes the first contribution decision in a sequential public goods game, and this decision is observed by the other group members before they make their own contribution decisions. By contrast, in leaderless groups, all group members make their contribution decisions simultaneously, without observing any other member’s contribution beforehand. Leaders can influence group outcomes by signaling commitment, setting a clear cooperative benchmark, and coordinating behaviors (Antonakis et al., 2022; Gächter et al., 2012; Gächter & Renner, 2018; Moxnes & Van der Heijden, 2003; Zhou et al., 2015). However, existing evidence suggests that leadership effectiveness is highly dependent on the institutional design of the selection process. Accordingly, our analysis focuses on evidence directly relevant to sequential public goods games and leader-selection institutions.

A key insight from the literature is that leading by example may operate through informational and reciprocal channels in sequential interactions (Arbak & Villeval, 2013; Hermalin, 1998; Potters et al., 2007; Stanca et al., 2009). The effectiveness of these channels depends on the leader-selection process and the institutional consequences of accepting or rejecting leadership. These factors influence both the informativeness of leadership behaviors and followers’ willingness to reciprocate.

Two challenges have emerged from the literature regarding this process. First, leadership mechanisms with low entry barriers do not always succeed in generating a leader (Arbak & Villeval, 2013; Haigner & Wakolbinger, 2010; Rivas & Sutter, 2011). When a selection mechanism fails to yield a leader, the resulting absence of leadership sends a strong negative signal, leading to pessimism among followers and lower cooperation (Xu et al., 2025). Second, when leadership is distributed among multiple individuals, coordination difficulties and diffused accountability may diminish its effectiveness.

We conceptualize leadership as the influence exerted by the first mover in a sequential public goods game and investigate how alternative leader-selection mechanisms address these challenges and affect voluntary contributions compared to a leaderless baseline, focusing on two main objectives.

First, we provide a systematic comparison of three institutional processes for leader selection: exogenous leadership, voluntary leadership, and delegated leadership following refusal. We implement four treatments. Treatment 1 (T1, control) employs a leaderless voluntary contribution mechanism (VCM). Treatment 2 (T2, Random Leadership; RL) randomly appoints one group member as the leader. To isolate the effect of individual consent from exogenous assignment, Treatment 3 (T3, Voluntary Leadership from a Random Candidate; VL-RC) allows a randomly selected candidate to accept or reject the leadership role; rejection results in no leader. Additionally, we introduce a novel mechanism, Treatment 4 (T4, Delegated Leadership by a Random Candidate; DL-RC), which also begins with a randomly selected candidate. If the candidate accepts, the game proceeds with a single leader. If the candidate refuses, the remaining three group members move first jointly, while the original candidate becomes the sole follower. This allows us to examine whether refusal yields different behavioral outcomes depending on whether it leads to no leadership or to a collective leadership structure.

Second, we compare fixed matching with random rematching to assess whether the effectiveness of each mechanism differs when stable-group membership and repeated-partner reputational incentives are present versus when such incentives are weakened by stranger matching.

Our experimental design employs a unified 2×4 framework that bridges and extends two strands of the existing literature. Prior studies have investigated standard leadership mechanisms under either random matching (Xu et al., 2025), which weakens stable repeated-partner reputational incentives, or fixed matching (Haigner & Wakolbinger, 2010; He & Zheng, 2024), which allows repeated interactions; however, they have not simultaneously varied leader-selection rules and matching protocols within a single experimental design.

Existing studies have investigated a wide range of sequential-move structures, including one-leader games and alternative move orderings such as fully sequential and multi-leader games (Eichenseer, 2023; Haigner & Wakolbinger, 2010; Helland et al., 2018). However, the behavioral implications of delegating first-mover responsibility following a candidate’s refusal—which explicitly transfers the leadership burden to the remaining group members—have not been studied. Our DL-RC mechanism enables us to evaluate the overall performance of this refusal-triggered delegation rule under both fixed and random matching. Because the refusal event and the three-first-mover structure are jointly generated by the mechanism, the design does not separately identify the independent effect of leader multiplicity. Therefore, we examine whether the complete DL-RC institutional arrangement affects cooperation and use realized-state comparisons to determine where its aggregate performance is concentrated. We hypothesize that reputational incentives under fixed matching encourage leadership and contributions. In contrast, random matching is likely to exacerbate free-riding behaviors when leadership is either declined or shared among multiple members.

Using this framework, we identify five main findings while distinguishing descriptive rankings from confirmatory inference. First, leadership does not uniformly increase contributions. DL-RC exhibits the lowest observed mean contribution overall, particularly under random rematching; however, the primary omnibus and Holm-adjusted model-based pairwise tests do not establish a general mechanism effect. Second, within VL-RC and DL-RC, observed contributions differ substantially across realized acceptance and refusal branches. These comparisons are conditional on endogenous candidate decisions and do not identify causal effects of leadership acceptance, refusal, leader absence, or leader multiplicity. Third, first movers generally contribute more than later movers in leader-present groups, and later-mover contributions are positively associated with observed first-mover contributions. Fourth, the endogenous mechanisms produce different realized group structures; the lower observed contribution in DL-RC is descriptively concentrated in the refusal-contingent three-first-mover branch. Finally, fixed matching has a higher observed mean contribution than random rematching, but the pooled difference is not statistically significant; the clearest within-mechanism matching contrast occurs in DL-RC.

The rest of this paper is organized as follows. Section 2 reviews the literature. Section 3 describes the experimental design, Section 4 presents the results, and Section 5 concludes.

## 2. Literature Review

The economic analysis of leadership as an institutional mechanism for addressing collective-action problems originates with Hermalin (1998), who conceptualizes “leading by example” as a sequential-move process in which a first mover coordinates beliefs and behaviors. In studies of experimental public goods games, leadership typically operates through two interrelated channels. The first is an informational channel: the leader’s contribution provides a benchmark that can shift followers’ expectations and serve as a credible signal of intent or commitment (Antonakis et al., 2022; Gächter et al., 2012; Pietraszewski, 2020; Potters et al., 2007). The second is a reciprocity channel: followers respond conditionally to the perceived cooperativeness of the leader, rewarding positive examples and punishing negative ones through their own contribution decisions (Arbak & Villeval, 2013; Stanca et al., 2009). The interaction between these two channels determines whether leadership functions as a catalyst for cooperation or as a trigger for collective free-riding.

The existing literature has increasingly explored the effects of selection mechanisms on leadership outcomes. For exogenous leadership—where the first mover is assigned randomly—the empirical evidence is mixed. Some studies report significant improvements in efficiency (Dannenberg, 2015; Levati et al., 2007; Moxnes & Van der Heijden, 2003), while others report minimal or statistically insignificant effects on cooperation (Gächter & Renner, 2018; Haigner & Wakolbinger, 2010). In contrast, endogenous leadership operates as a costly signal, reflecting a leader’s willingness to bear the burden of leadership and to forgo the advantage associated with moving second (Eichenseer, 2023). Consequently, some studies find that voluntary leadership promotes higher levels of cooperation than externally imposed leadership (Dannenberg, 2015; Haigner & Wakolbinger, 2010). This variability suggests that the informational and reciprocal significance of leadership signals depends on the underlying selection mechanisms. The importance of institutional credibility is further emphasized in applied contexts such as climate cooperation. Helland et al. (2018) demonstrate that the effectiveness of conditional commitments is fundamentally linked to their credibility and to the perceived fairness of the institutional arrangement.

A second institutional dimension concerns the allocation of responsibility and influence in contexts of shared leadership. Empirical behavioral evidence indicates that distributing decision-making responsibility may dilute accountability and foster increased self-interested behavior compared to situations involving a single, clearly accountable decision maker (Charness & Jackson, 2009). In public goods games, collective leadership may similarly inhibit prosocial behavior, as group leaders tend to exhibit lower levels of cooperation than individual role models (Zhou et al., 2015). Supporting this perspective, Xu et al. (2025) demonstrate that unrestricted voluntary leadership (VL)—which often results in multiple leaders—can yield suboptimal outcomes due to coordination difficulties and diminished accountability.

We extend this body of the literature by examining a unique case in which shared leadership is not voluntarily adopted but is institutionally mandated following an act of refusal. Unlike settings where multiple leaders emerge voluntarily (Xu et al., 2025), the DL-RC framework investigates a specific institutional response to refusal, wherein the remaining group members are assigned collective first-mover responsibility. This design allows us to evaluate whether the refusal-triggered delegated arrangement is associated with different contribution outcomes. However, because refusal and the multiple first-mover structure occur simultaneously, the design does not isolate responsibility diffusion from alternative explanations such as negative inferences from refusal, coordination difficulties, or concerns about the legitimacy of the delegated roles. Therefore, we interpret the realized DL-RC branches as conditional outcomes generated by the complete institutional package rather than as direct evidence of any single psychological mechanism.

Furthermore, this study contributes to the literature by examining how leader-selection rules interact with matching protocols. Previous studies have typically evaluated particular leadership mechanisms within a single matching environment. For example, fixed matching allows stable-group membership and thereby creates scope for repeated-interaction, reciprocal, and reputational incentives (He & Zheng, 2024), whereas random rematching weakens stable-group-specific relationships while preserving the observability of leadership behavior within each period (Xu et al., 2025). By implementing the same set of leader-selection mechanisms under both protocols, our design provides a unified framework for assessing whether their behavioral performance varies with the availability of stable repeated-partner incentives. This comparison motivates our hypotheses concerning the relative performance of RL, VL-RC, and DL-RC under fixed matching and random rematching.

## 3. Experimental Design

### 3.1. Basic Setting

The experimental design is based on the voluntary contribution mechanism (hereafter, VCM). Throughout the experiment, we define a leader operationally as a first mover in the contribution sequence. In leader-present groups, the leader, or leaders in the case of delegated leadership, choose their contributions before the later mover(s), and these first-mover contributions are revealed to the later mover(s) before they make their own contribution decisions. In leaderless groups, all four participants make contribution decisions simultaneously, and no participant observes another group member’s contribution before deciding. In each session, participants are assigned to groups of four and engage in interactions over 10 periods. Depending on the treatment, group composition either remains constant throughout the experiment (fixed matching) or is randomly re-matched in each period (random matching).

In each period, subjects are endowed with 20 tokens. Although the sequence of moves varies across treatments, the payoff structure is identical in all treatments. Participants decide how to distribute their endowment between a private account and a public account. The Marginal Per Capita Return (MPCR) from the public account is 0.4, meaning every token contributed to the public pool yields a total of 1.6 tokens shared equally by the group. Let $c_{it}$ denote the individual *i*’s contribution to the public account in period *t*, restricted to an integer that satisfies $0\leq c_{it}\leq 20$. Let $\pi_{it}$ denote individual *i*’s earnings from their private account and the public account in period *t*, given the contributions of all group members ${\left\{c_{jt}\right\}}_{j=1}^{4}$:

$$\pi_{it}=20-c_{it}+\frac{1.6\times \sum_{j=1}^{4}c_{jt}}{4}.$$

### 3.2. Matching Protocols and Leader-Selection Mechanisms

The experiment employs a 2×4 factorial design, interacting two matching protocols with four distinct leader-selection mechanisms, yielding a total of eight treatments.

**Matching Protocols.** To investigate the interaction between institutional mechanisms and repeated-interaction incentives, we implement two matching rules.^1^

1. **Fixed Matching:** Group membership is fixed for all periods. This protocol creates stable-group-level repeated interaction and may allow for reciprocal or reputational incentives within the group.
2. **Random Rematching:** Groups are randomly re-formed in each period. We use this protocol to weaken stable-group-specific reputational incentives and repeated interaction within the same group. Because the same participants remain in the experiment for 10 periods and may learn from previous outcomes, this protocol should not be interpreted as a sequence of fully independent one-shot games. Rather, it approximates stranger matching by preventing stable-group membership across periods.

**Leader-Selection Mechanisms.** Within each matching protocol, we implement the following four distinct mechanisms. These mechanisms differ in their selection processes (exogenous vs. endogenous) and in the default outcome when the leadership role is not accepted.

1. **Control (Baseline):** Subjects participate in a standard VCM. All group members make their contribution decisions independently and simultaneously.
2. **RL (Random Leadership):** One randomly selected group member is exogenously appointed as the leader by the computer. The game proceeds sequentially: the leader first decides on their contribution, which is then revealed to the three followers. After observing the leader’s decision, followers decide simultaneously on their respective contributions.
3. **VL-RC (Voluntary Leadership by the Randomly Selected Candidate):** One group member is randomly selected as a candidate and offered the leadership role.
  - If the candidate accepts, they become the sole voluntary leader and contribute first (identical to the RL treatment).
  - If the candidate declines, the leadership position is dissolved. The group reverts to the standard VCM (control), where all members contribute simultaneously.
4. **DL-RC (Delegated Leadership by the Randomly Selected Candidate):** One group member is randomly selected as a candidate and offered the leadership role.
  - If the candidate accepts, the procedure follows the same one-leader sequence as in the accepted branch of VL-RC: the candidate becomes the sole voluntary leader and contributes first.
  - If the candidate declines, leadership responsibility is delegated to the other three group members. In this sub-game, the three delegated leaders contribute first, while the original candidate becomes the sole follower and decides only after observing the delegated leaders’ contributions.

In all treatments, subjects receive anonymous feedback on group members’ roles, contributions, and payoffs at the end of each period.

Our experimental design is structured to bridge and extend the specific gaps in the literature left by Xu et al. (2025) and He and Zheng (2024).

The existing literature has comprehensively examined the standard leadership mechanisms (control, RL, VL-RC) under distinct matching protocols. While Xu et al. (2025) investigated these mechanisms under a random-rematching protocol that weakens stable repeated-partner incentives, He and Zheng (2024) provided a counterpart analysis under a fixed-matching protocol that allows reputation building within stable groups.

Despite these contributions, the strategic implications of delegation remain unexplored—particularly the scenario where rejecting leadership explicitly shifts the coordination burden onto other group members. To fill this gap, we introduce a novel DL-RC (delegated leadership) mechanism and implement it across both matching protocols to establish a complete 2 × 4 comparative framework. The design enables us to identify the overall effect of assignment to the DL-RC institutional condition relative to the control, RL, and VL-RC and to examine how its performance varies across different matching environments.

### 3.3. Hypotheses

Following the game-theoretic framework of Hermalin (1998), we posit that leaders facilitate public goods provision through signaling and reciprocity. We hypothesize that the varying effectiveness of our mechanisms originates from differences in the selection process. Specifically, the selection rule dictates whether the act of leading serves as an informative signal of the leader’s cooperative intent.

We view the selection mechanism as shaping how informative leadership behavior is to followers. Upon observing the leader’s contribution, followers update their beliefs regarding the leader’s type (altruistic vs. self-interested) and the group’s cooperative potential. However, the clarity of this signal depends critically on the counterfactual consequences of declining the leadership role.

The experimental design examines the effects of alternative leader-selection and matching institutions on observable role choices and contribution behavior. Although we do not directly measure psychological mediators such as beliefs, intentions, or perceived accountability, mechanisms such as signaling, responsibility allocation, and reputation provide theoretically grounded channels that inform our testable predictions. Consequently, our empirical tests focus on differences in leadership acceptance, conditional contribution behavior, and group outcomes across institutional conditions.

Table 1 summarizes the structural differences and strategic properties of the three active leadership mechanisms:

We start by discussing the key design features of the treatments with respect to the number of leaders. DL-RC is the only mechanism that generates multiple first movers (Fact F1). While RL is an exogenous leader-selection mechanism that guarantees a single leader in every period, VL-RC and DL-RC are endogenous, as the randomly selected candidate may either accept or decline the leadership role. In VL-RC, a rejection results in a leaderless group (Fact F2). Conversely, DL-RC precludes a leaderless outcome by shifting the first-mover responsibility to the remaining group members upon the candidate’s rejection, thereby potentially creating multiple leaders (Fact F1).

Table 1 further highlights whether a mechanism respects individual willingness not to lead (Attribute A1). VL-RC and DL-RC incorporate this feature by allowing the selected candidate to decline the role, whereas RL imposes leadership exogenously, regardless of the subject’s underlying motivation.

Candidates who voluntarily accept roles in VL-RC and DL-RC effectively select themselves into the first-mover position. This observable selection is expected to make their subsequent contributions more informative to followers than those of randomly appointed leaders, thereby potentially enhancing the value of their actions as signals of cooperative intent. In contrast, leaders in RL are randomly assigned and may lack intrinsic motivation to promote group cooperation.

A distinctive feature of DL-RC is that rejection by the candidate induces multiple delegated leaders. In this multiple-leader sub-game, each delegated leader may expect the others to shoulder the responsibility for public good provision, giving rise to a free-riding incentive among delegated leaders (Effect E1).

Finally, VL-RC is the only mechanism in which the absence of a leader conveys information. However, this signal is inherently noisy (Effect E3): a leaderless outcome arises solely because the single randomly selected candidate refused, which does not preclude the possibility that other group members would have been willing to lead. Consequently, the no-leader state in VL-RC provides an imprecise signal of low cooperative intent.

The hypothesized behavioral implications summarized in Table 1 may also depend on the matching protocol. Under fixed matching, stable-group membership creates scope for repeated-interaction, reciprocal, and reputational incentives. These incentives may strengthen the behavioral significance of voluntary acceptance (E2) and may limit the adverse consequences associated with shared first-mover responsibility in the refusal-triggered branch of DL-RC (E1). Under random rematching, stable-group-specific incentives are weakened, although leadership choices and contributions remain observable within each period. In this environment, voluntary leadership signals may carry less influence across periods, while the refusal-triggered multiple-first-mover arrangement may be more vulnerable to coordination difficulties or reduced individual accountability.

Based on these considerations, we hypothesize the following efficiency ranking. VL-RC combines positive selection (E2) with the absence of multiple-leader free-riding (E1), making it a strong candidate for the most effective mechanism. RL suffers from the lack of leader motivation (weak E2). DL-RC represents an intermediate case: while it screens for willingness in the acceptance branch (E2), it entails the structural risk of multiple forced leaders (E1) following rejection. We expect this disadvantage to be mitigated under fixed matching, where reputational incentives discourage rejection and thereby reduce the inefficient outcome.

### 3.4. Procedure

A total of 256 college students from Tsinghua University participated in the experiment between April and July 2020. The experiment was conducted as a controlled online laboratory experiment during the COVID-19 pandemic. Participants were recruited from the university subject pool, signed an informed consent form before participation, and were allowed to participate only once. This study followed standard ethical protocols and was approved by the Institutional Review Board (IRB) prior to data collection.

We implemented a between-subjects design with eight experimental conditions, corresponding to four leader-selection mechanisms crossed with two matching protocols. Each experimental condition included 32 participants and was conducted in two sessions of 16 participants each. Thus, each session consisted of four groups of four participants, and each participant made decisions over 10 periods.

Each session implemented one experimental condition. Within each session, group assignment and role assignment were implemented by the z-Tree program. In the fixed-matching condition, participants were randomly assigned to groups of four at the beginning of the first period, and group membership then remained unchanged for all 10 periods. In the random-rematching condition, groups of four were randomly re-formed in each period. In the RL treatment, one group member was randomly assigned as the first mover in each period. In the VL-RC and DL-RC treatments, one group member was randomly selected as the leader candidate in each period and then decided whether to accept the leadership role. Participants were informed of the relevant matching and role-assignment procedures before making decisions.

Participants signed in simultaneously at the scheduled session time and completed the controlled online laboratory experiment remotely using z-Tree version 4.1.6 (Fischbacher, 2007) under online supervision by the experimenter. The experimenter guided participants through the instructions in an online meeting. Participants were instructed not to communicate with one another during the experiment, and any questions were answered privately by the experimenter. At the end of each period, participants received anonymous feedback on current-period roles, contributions, total group contribution, and payoffs. Role histories were not displayed to participants. No participants were excluded from the analysis, and there was no attrition or technical failure during the experiment.

Each session lasted approximately 20 min. Earnings were accumulated over 10 periods at an exchange rate of 1 token = 0.05 CNY. The average payment received was about 17.92 CNY per subject, including a 5 CNY show-up fee, which is comparable to standard online payment levels in mainland China.

## 4. Results

This section presents the experimental findings, structured as follows. We begin by analyzing the average contribution levels across all treatments, followed by a comparison between groups with and without a designated leader. Subsequently, we explore the behavioral dynamics inherent in leader–follower interactions. Additionally, we investigate the strategic incentives for free-riding within endogenous leadership mechanisms. Finally, we assess the impact of matching protocols by comparing outcomes from fixed and random matching rules.

### 4.1. Comparison of Average Contributions

**Result 1**. Leadership does not produce a uniform increase in contributions across different mechanisms. DL-RC exhibits the lowest observed mean contribution overall, especially under random rematching; however, the primary confirmatory comparisons do not reveal a statistically significant general effect of the mechanism.

Table 2 reports average individual contributions across the four leader-selection mechanisms. The overall averages are 7.052 in control, 7.813 in RL, 7.698 in VL-RC, and 5.578 in DL-RC. Thus, DL-RC has the lowest descriptive average contribution overall.

Table 3 presents the results separately by matching protocol. In fixed matching, the average contributions are similar in all four mechanisms: 8.038 in control, 7.794 in RL, 8.053 in VL-RC, and 7.044 in DL-RC. Under random rematching, the corresponding averages are 6.066, 7.831, 7.344, and 4.113. The largest descriptive differences, therefore, arise under random rematching.

The mixed-effects estimates are reported in Appendix B Table A1. The overall mechanism test is not statistically significant ($\chi^{2}\left(3\right)=1.58$, p=0.665). The protocol-specific omnibus tests are also not statistically significant under fixed matching ($\chi^{2}\left(3\right)=0.11$, p=0.990) or random rematching ($\chi^{2}\left(3\right)=4.05$, p=0.256). In addition, the mechanism-by-matching interaction is not statistically significant ($\chi^{2}\left(3\right)=0.65$, p=0.885).

Appendix B Table A2 reports all six pairwise mechanism contrasts for the overall sample and separately within each matching protocol, with Holm correction applied within each comparison family. None of the primary model-based contrasts is statistically significant at the 5% level. The largest protocol-specific estimate occurs under random rematching, where DL-RC is 3.719 tokens lower than RL (unadjusted p=0.066; Holm-adjusted p=0.394).

Appendix B Table A3 reports a session-level HC2 sensitivity analysis. The mechanism-by-matching interaction remains statistically insignificant (p=0.678), and most pairwise contrasts are also insignificant. Under random rematching, however, DL-RC is estimated to be 3.231 tokens lower than VL-RC (unadjusted p=0.002; Holm-adjusted p=0.011). Taken together, the lower observed mean in DL-RC is a consistent descriptive pattern supported by this one session-level sensitivity comparison, but it is not established as a broadly robust treatment effect by the primary omnibus or Holm-adjusted model-based pairwise analyses.

**Figure 1:**
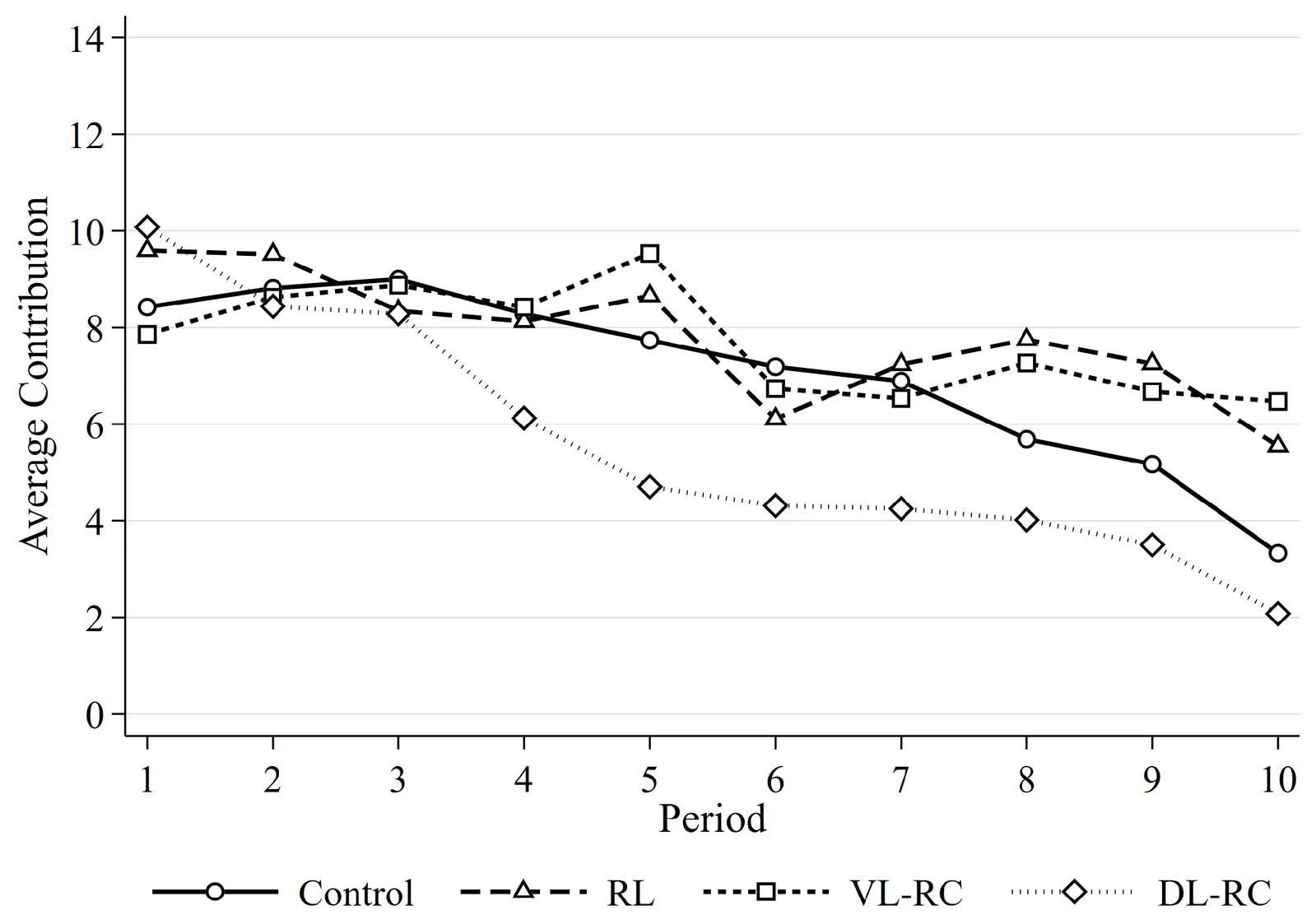
Average contributions over periods by treatment.

Figure 1 presents average individual contributions across periods, pooling the two matching protocols. Contributions display an overall downward tendency in all four mechanisms, although the trajectories fluctuate over time. DL-RC begins at the highest descriptive level in Period 1, declines sharply after Period 3, and has the lowest average contribution from Period 4 onward. The mixed-effects trajectory model rejects the joint null that the mechanism-by-period and mechanism-by-matching-by-period interaction terms are zero ($\chi^{2}\left(54\right)=128.36$, p<0.001, see Appendix B Table A4). This result indicates that contribution paths vary jointly across mechanisms and matching protocols, but it does not identify a specific pairwise difference in trajectories.

### 4.2. Comparison of Average Contributions Between Groups with and Without a Leader

**Result 2**. Within the endogenous mechanisms, observed contributions differ significantly between the realized acceptance and refusal branches. In VL-RC, the accepted one-leader branch exhibits higher observed contributions than the refusal-contingent leaderless branch. In DL-RC, the refusal-contingent three-first-mover branch shows lower observed contributions than the accepted one-leader branch. Since these branches are endogenously selected, the comparisons are conditional and not causal.

The comparisons in this subsection are conditional on realized leadership states. In VL-RC and DL-RC, these states are generated by the selected candidate’s decision to accept or decline the leadership role and are therefore endogenously selected. The results describe how contributions differ across the branches generated by each mechanism, but they do not identify the causal effects of accepting leadership, declining leadership, remaining leaderless, or having one rather than three first movers.

Table 4 presents average individual contributions separately by the realized leadership state and matching protocol. Within VL-RC, average contributions are higher when the selected candidate accepts the leadership role than when the candidate declines and the group remains leaderless. The corresponding averages are 12.043 versus 2.371 under fixed matching and 11.038 versus 3.829 under random rematching.

Within DL-RC, groups in the accepted one-leader branch contribute more than groups in the refusal-contingent three-leader branch. The corresponding averages are 10.163 versus 5.785 under fixed matching and 7.750 versus 3.056 under random rematching.

All four comparisons remain statistically significant after Holm correction (p<0.001). However, these realized states are generated by endogenous acceptance or refusal decisions. The comparisons therefore describe conditional differences between observed branches and should not be interpreted as causal effects of leadership acceptance, leadership absence, or leader multiplicity. In particular, the three-leader DL-RC branch combines the candidate’s refusal, a change in the contribution sequence, and the assignment of multiple first movers.

### 4.3. Comparison of Leader–Follower Interactions

**Result 3**. In a leader-present environment, first movers contribute more on average than later movers. After accounting for the dependence between decisions made within the same group-period and correcting for multiple comparisons, the first-mover versus later-mover gap remains statistically significant under fixed matching only in the accepted one-leader branch of DL-RC. In contrast, under random rematching, this gap is statistically significant in all four realized leadership states. Across leader-present states, later-mover contributions are also positively correlated with the observed mean contribution of the first mover or movers in the same group-period.

Table 5 reports descriptive individual contributions by role and realized leadership state. Under fixed matching, first movers contribute an average of 10.300 tokens in RL and 14.191 tokens in the accepted VL-RC branch, compared with 6.958 and 11.326 tokens among later movers, respectively. Within DL-RC, the corresponding first- and later-mover averages are 15.130 and 8.507 in the accepted one-leader branch, but only 6.450 and 3.789 in the refusal-contingent three-leader branch.

A similar descriptive ordering appears under random rematching. First movers contribute 11.225 tokens in RL and 16.231 tokens in the accepted VL-RC branch, compared with 6.700 and 9.308 tokens among later movers. Within DL-RC, first- and later-mover contributions average 13.667 and 5.778 in the accepted one-leader branch, compared with 3.591 and 1.452 in the refusal-contingent three-leader branch.

Since first- and later-mover decisions are generated within the same group-period, formal inference is based on the within-group-period contribution gap, defined as the mean contribution of the first mover or movers minus the mean contribution of the later mover or movers. This approach directly compares the decisions generated within the same group-period rather than treating the two roles as independent samples.

Under fixed matching, the estimated gap is positive in all four realized leadership states. The adjusted gaps are 3.347 tokens in RL (95% CI [0.055,6.639]; Holm-adjusted p=0.093), 3.960 tokens in the accepted VL-RC branch (95% CI [0.512,7.409]; Holm-adjusted p=0.073), 6.875 tokens in the accepted one-leader DL-RC branch (95% CI [3.236,10.513]; Holm-adjusted p<0.001), and 2.568 tokens in the refusal-contingent three-leader DL-RC branch (95% CI [−0.783,5.918]; Holm-adjusted p=0.133). After Holm correction, only the accepted one-leader DL-RC gap remains statistically significant under fixed matching.

Under random rematching, the estimated gaps are 4.528 tokens in RL (95% CI [3.525,5.531]), 6.911 tokens in the accepted VL-RC branch (95% CI [5.456,8.366]), 8.057 tokens in the accepted one-leader DL-RC branch (95% CI [5.905,10.209]), and 2.095 tokens in the refusal-contingent three-leader DL-RC branch (95% CI [0.951,3.239]). All four gaps remain statistically significant after Holm correction (p<0.001; Appendix B Table A8).

The lower later-mover contribution in the accepted one-leader DL-RC branch is not explained by weaker first-mover contributions. Under fixed matching, accepted first movers contribute 14.191 tokens in VL-RC and 15.130 tokens in the accepted one-leader DL-RC branch. The adjusted DL-RC-minus-VL-RC difference is 1.781 tokens (95% CI [−2.120,5.681]; Holm-adjusted p=0.635). Under random rematching, the corresponding means are 16.231 and 13.667, and the adjusted difference is −3.099 tokens (95% CI [−9.176,2.977]; Holm-adjusted p=0.635). Thus, accepted first movers do not contribute significantly less in DL-RC than in VL-RC under either matching protocol.

More importantly, the focused later-mover analysis shows that the same observed first-mover contribution is followed by less cooperation in DL-RC. After controlling for the observed first-mover contribution, matching protocol, and period fixed effects, later movers in the accepted one-leader DL-RC branch contribute 3.015 tokens less than those in the accepted VL-RC branch (SE=1.048, 95% CI [−5.495,−0.536], p=0.024). A one-token increase in the observed first-mover contribution is associated, on average, with a 0.521-token increase in the later-mover contribution (SE=0.098, 95% CI [0.289,0.752], p=0.001). Taken together, these estimates indicate that the DL-RC environment is less effective at translating a given first-mover contribution into subsequent cooperation.

The interaction analysis further identifies where this weaker transmission arises. Under fixed matching, a one-token increase in the observed first-mover contribution is associated with a 0.756-token increase in later-mover contributions in VL-RC, but only a 0.356-token increase in the accepted one-leader DL-RC branch. The DL-RC-minus-VL-RC difference in response slopes is −0.400 (SE=0.137, 95% CI [−0.723,−0.077]; unadjusted p=0.022; Holm-adjusted p=0.044). Later movers therefore respond significantly less strongly to the first-mover contribution in DL-RC under fixed matching.

Under random rematching, the corresponding response slopes are 0.353 in VL-RC and 0.472 in DL-RC. Their difference is 0.119 (SE=0.116, 95% CI [−0.156,0.393]; Holm-adjusted p=0.340). The difference between the fixed- and random-matching slope contrasts is itself statistically significant (difference =0.519, SE=0.127, 95% CI [0.218,0.819], p=0.005). The weaker follower responsiveness in DL-RC is therefore concentrated in the fixed-matching environment.

The broader later-mover analysis yields the same central pattern that first-mover behavior is transmitted to subsequent contributors. Using all leader-present states, a one-token increase in the observed mean contribution of the first mover or movers is associated with a 0.526-token increase in the later-mover contribution (SE=0.029, 95% CI [0.470,0.583], p<0.001; Appendix B Table A11). This result confirms a strong positive relationship between the contribution signal sent by first movers and the contribution subsequently selected by later movers.

The distribution of contributions further clarifies where the lower observed contributions in DL-RC are concentrated. Defining a low first-mover contribution as no more than 5 of the 20 available tokens, the share of low first-mover observations under fixed matching is 54.12% in DL-RC but only 10.64% in VL-RC. Under random rematching, the corresponding shares are 73.04% and 7.69%. Thus, low first-mover contributions occur substantially more often in DL-RC, particularly in the refusal-contingent three-first-mover branch. Along with the lower follower responsiveness estimated under fixed matching, these results reveal two associated behavioral patterns: more frequent low first-mover contributions in the refusal-contingent branch and weaker transmission of a given first-mover contribution in the accepted branch. However, they do not establish the unmeasured psychological mechanisms underlying these patterns.

**Figure 2:**
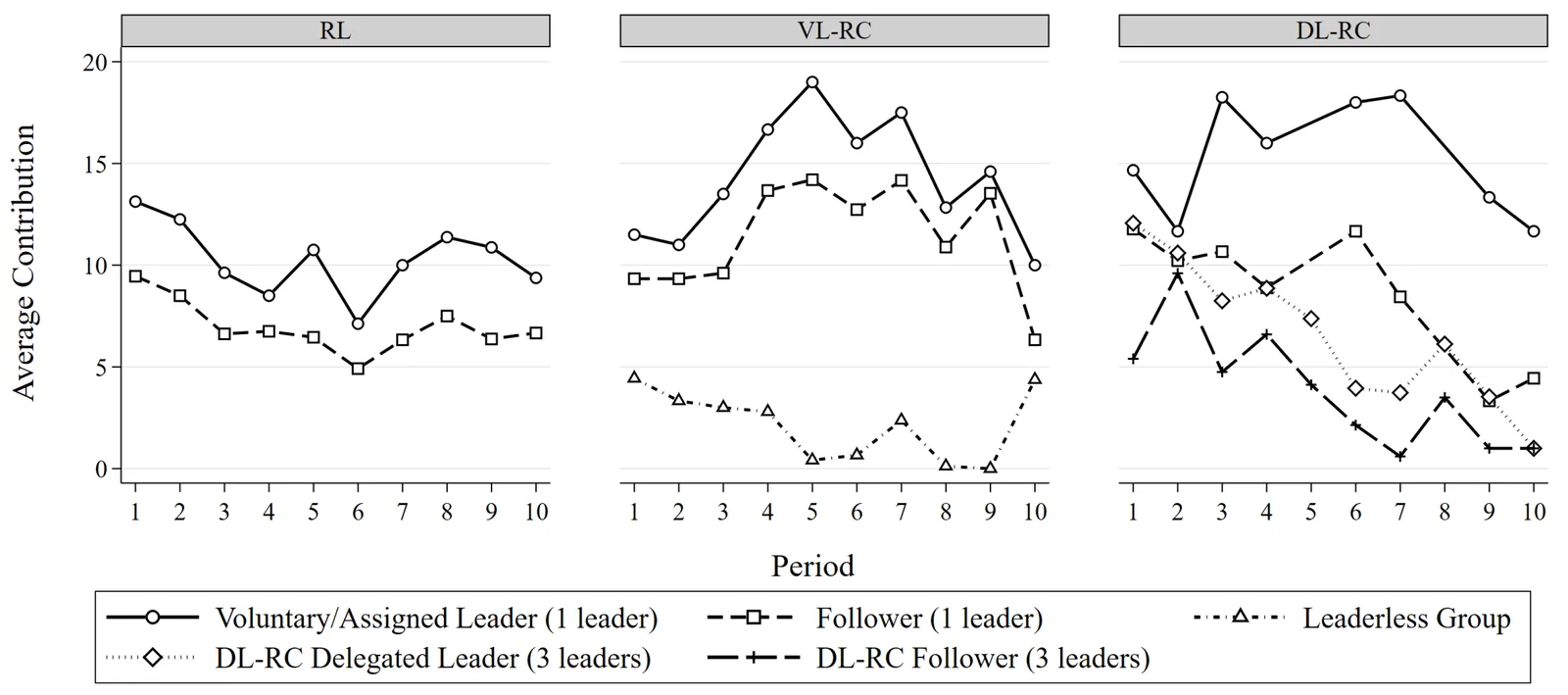
Average contribution over periods by role (fixed).

**Figure 3:**
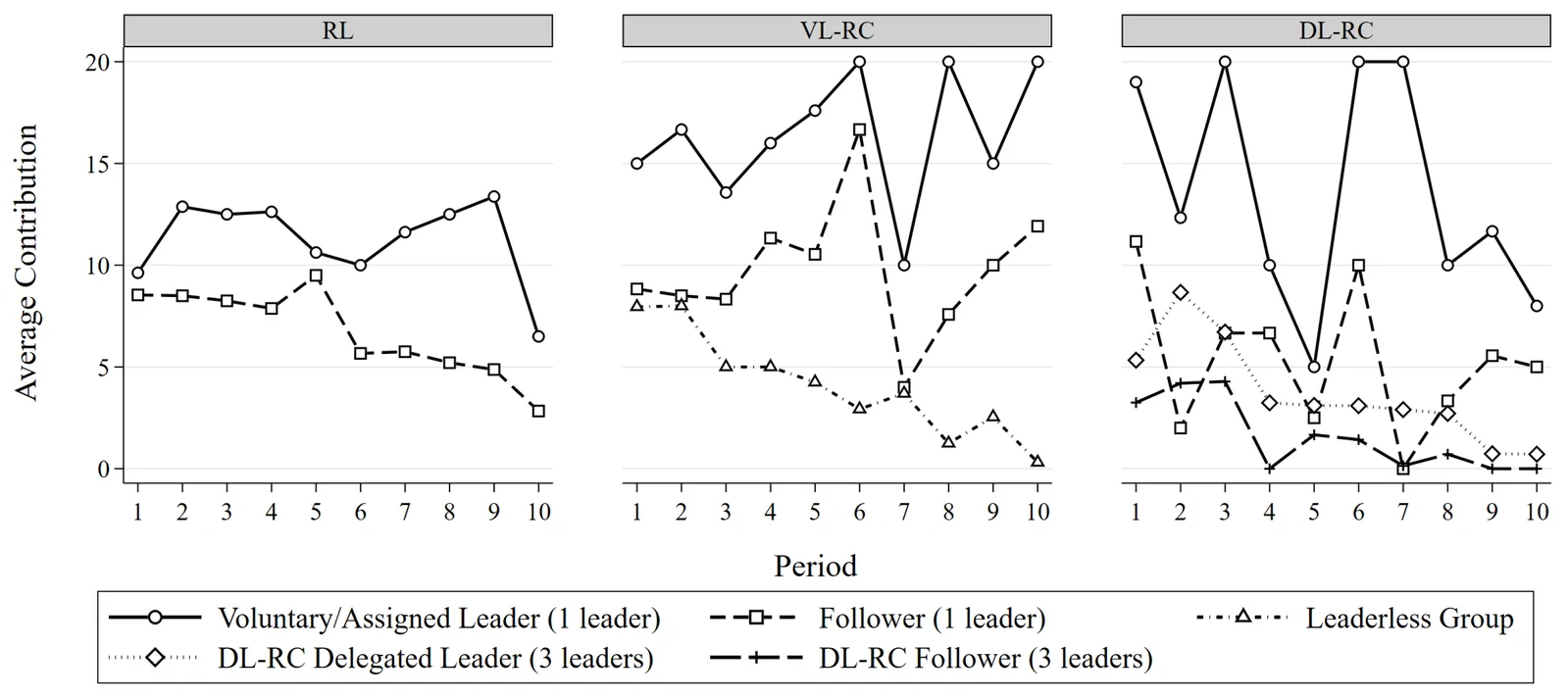
Average contribution over periods by role (random).

Figure 2 and Figure 3 present the contribution trajectories by mover role under fixed matching and random rematching, respectively. Across most periods, first movers contribute more than later movers. The figures also show that the low DL-RC contributions are concentrated in the refusal-contingent three-leader branch, where both first- and later-mover contributions remain substantially below those in the accepted one-leader branch.

### 4.4. Discussion of Endogenous Leadership

**Result 4**. Endogenous leadership mechanisms generate different realized group structures. DL-RC produces the refusal-contingent three-first-mover structure more frequently than VL-RC produces a leaderless structure, and the lower observed contribution in DL-RC is descriptively concentrated in that refusal-contingent branch. These realized-state comparisons remain conditional on endogenous candidate decisions.

Table 6 presents candidate acceptance rates and the resulting leadership structures. Under fixed matching, candidates accept in 47 of 80 VL-RC candidate-periods, corresponding to an acceptance rate of 58.75%, compared with 23 of 80 candidate-periods, or 28.75%, in DL-RC. Under random rematching, the corresponding acceptance rates are 48.75% in VL-RC and 22.50% in DL-RC.

We formally compare candidate acceptance using mixed-effects logistic regression models that account for repeated candidate decisions. In the full candidate-period model, the adjusted probability of acceptance is 57.06% in VL-RC and 27.11% in DL-RC. The difference between DL-RC and VL-RC is therefore −29.95 percentage points (SE=8.38, 95% CI [−46.37,−13.53], p<0.001). Candidates are considerably less willing to enter the voluntary one-leader branch when refusal transfers first-mover responsibility to the other three group members.

The mechanism difference does not vary significantly across matching protocols, $\chi^{2}\left(1\right)=0.321$, p=0.571. Nevertheless, the protocol-specific estimates differ in precision. Under fixed matching, the adjusted acceptance probabilities are 62.97% in VL-RC and 30.53% in DL-RC, resulting in a difference of −32.44 percentage points (SE=16.59, 95% CI [−64.95,0.07]; Holm-adjusted p=0.0505). Under random rematching, the adjusted probabilities are 48.28% and 25.21%, respectively, yielding a difference of −23.07 percentage points (SE=9.49, 95% CI [−41.66,−4.48]; Holm-adjusted p=0.030).

A session-level sensitivity analysis using HC2 standard errors maintains the negative direction of all three comparisons but yields substantially wider confidence intervals due to the analysis including only two sessions per mechanism-by-matching cell. In this sensitivity analysis, the random-rematching difference remains statistically significant after Holm correction, whereas the overall and fixed-matching estimates are imprecisely estimated. The complete mixed-effects and session-level results are reported in Appendix B Table A13 and Table A14.

The differences in acceptance decisions directly result in distinct realized group structures. Under fixed matching, VL-RC produces one voluntary first mover in 58.75% of group-periods and remains leaderless in the remaining 41.25%. By contrast, DL-RC produces one voluntary first mover in only 28.75% of group-periods and enters the three-delegated-first-mover branch in 71.25% of group-periods. Consequently, the average number of first movers per group-period is 0.588 in VL-RC but 2.425 in DL-RC.

The same structural contrast is observed under random rematching. VL-RC produces one voluntary first mover in 48.75% of group-periods and no first mover in 51.25%. DL-RC produces one voluntary first mover in 22.50% of group-periods and three delegated first movers in 77.50%, resulting in an average of 2.55 first movers per group-period.

**Figure 4:**
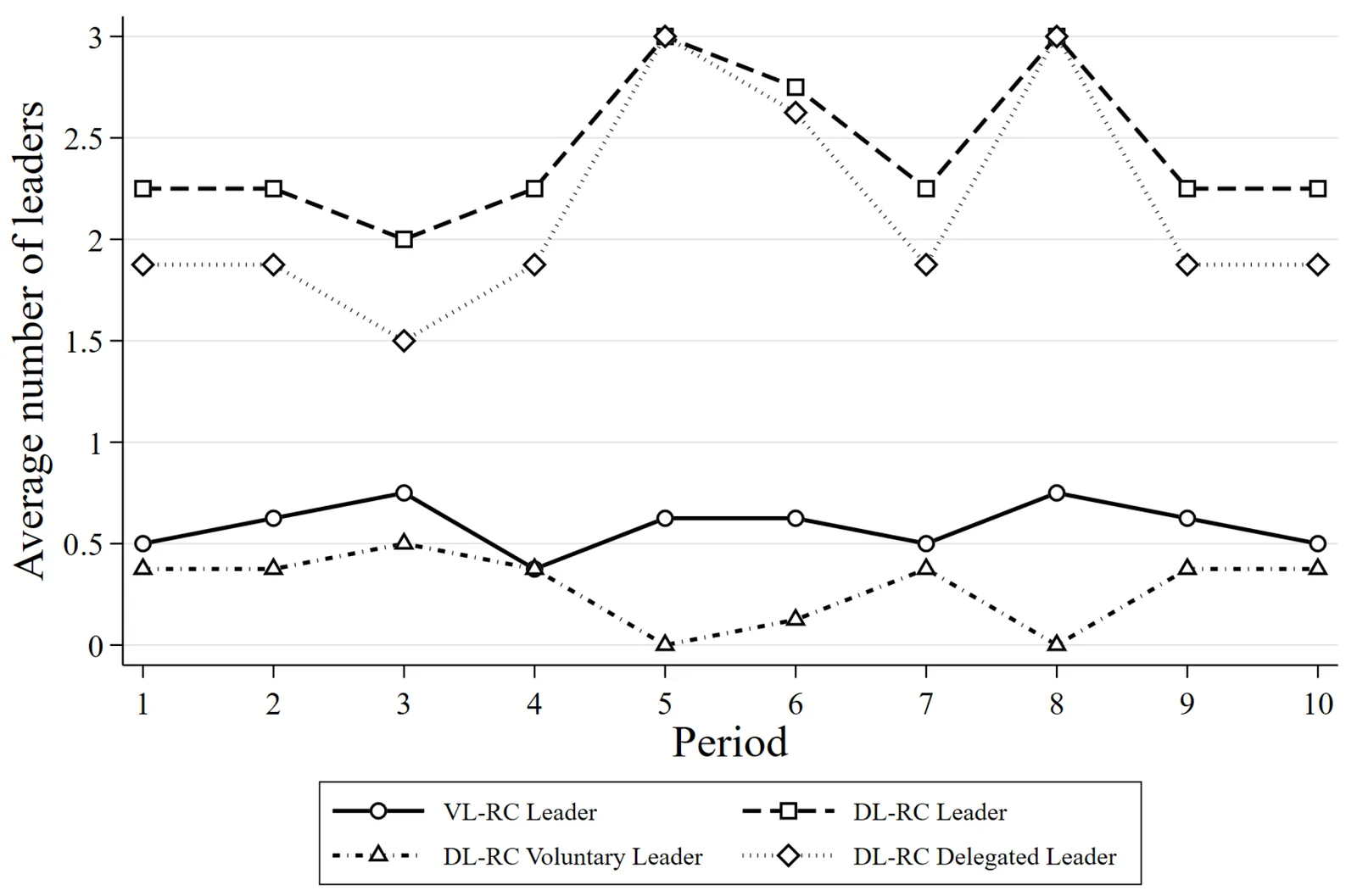
Average number of leaders over periods by treatment (fixed).

**Figure 5:**
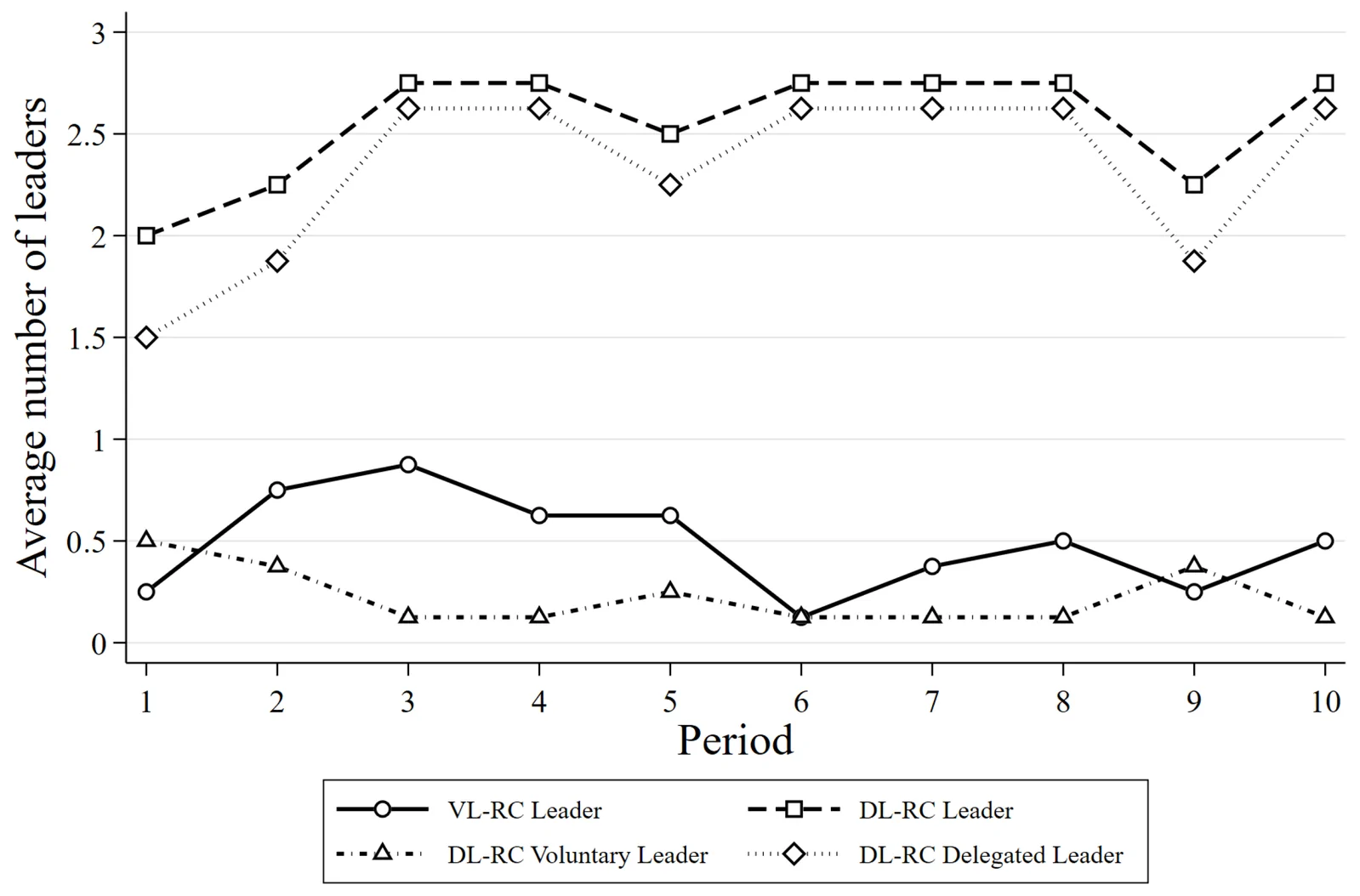
Average number of leaders over periods by treatment (random).

Figure 4 and Figure 5 illustrate the period-by-period development of these structures. In VL-RC, the average number of total first movers remains below one because a candidate’s refusal results in the group having no first mover. In DL-RC, the average number of total first movers generally exceeds two, as candidate refusal triggers the three-delegated-first-mover branch. The figures also indicate that the majority of first movers observed in DL-RC are delegated rather than voluntary.

These structural findings clarify the origin of the realized branches examined in Results 2 and 3. The low-contribution three-first-mover branch is not an occasional outcome within DL-RC; rather, it represents the mechanism’s modal realized state under both matching protocols. However, the current design does not independently manipulate the refusal event and the number of first movers. Therefore, the evidence from Result 4 establishes that DL-RC generates a substantially different acceptance pattern and realized role structure.

### 4.5. Comparison Between Fixed Matching and Random Matching

**Result 5**. Fixed matching exhibits a higher observed overall mean contribution than random rematching; however, the pooled difference is not statistically significant. The clearest statistically significant within-mechanism matching contrast is observed in DL-RC.

Table 7 first reports the overall comparison and then separates group-periods according to whether a leader is present. Across the complete sample, the average group-period contribution is 7.732 under fixed matching and 6.338 under random rematching. The mixed-effects model estimates a fixed-minus-random difference of 1.394 tokens, but this difference is not statistically significant (SE=1.212, 95% CI [−0.981,3.768], p=0.250). Adding leader-selection-mechanism fixed effects leaves the estimate unchanged (SE=1.257, 95% CI [−1.070,3.858], p=0.268).

Conditional on the realized leadership structure, the descriptive fixed-matching advantage remains visible but is not statistically significant. Among leader-present group-periods, the adjusted fixed-minus-random difference is 1.231 tokens (SE=1.183, 95% CI [−1.087,3.549]; raw p=0.298; Holm-adjusted p=0.596). Among leaderless group-periods, the corresponding estimate is 1.264 tokens (SE=2.013, 95% CI [−2.682,5.210]; raw p=0.530; Holm-adjusted p=0.596). Thus, the data do not support a general conclusion that fixed matching increases contributions either whenever a leader is present or whenever no leader emerges.

Table 8 examines the matching comparison within a fixed leader-selection mechanism. Among leader-present group-periods, contributions are virtually identical across matching protocols in RL. The estimated fixed-minus-random difference is −0.038 tokens (SE=2.692, 95% CI [−5.313,5.238]; Holm-adjusted p=1.000). In the realized leader-present branch of VL-RC, the corresponding estimate is 0.428 tokens (SE=2.800, 95% CI [−5.060,5.915]; Holm-adjusted p=1.000).

A different pattern emerges within DL-RC. Contributions average 7.044 under fixed matching and 4.113 under random rematching. The adjusted fixed-minus-random difference is 2.931 tokens (SE=0.911, 95% CI [1.145,4.717]; raw p=0.001; Holm-adjusted p=0.004). By contrast, neither of the leaderless comparisons is statistically significant. The estimated difference is 1.972 tokens in control (SE=2.040, 95% CI [−2.027,5.971]; Holm-adjusted p=0.668) and 0.583 tokens in the realized leaderless branch of VL-RC (SE=3.893, 95% CI [−7.048,8.213]; Holm-adjusted p=0.881).

These estimates indicate that the observed matching-protocol difference is not a general feature of leader-present or leaderless groups. Instead, the clearest difference is concentrated within the complete DL-RC institutional environment. Because DL-RC combines candidate acceptance or refusal, the resulting first-mover assignment, and the associated role structure, this comparison does not isolate a single behavioral mechanism through which fixed matching affects contributions.

We next examine whether the DL-RC difference is concentrated among first movers or later movers. Table 9 reports role-specific comparisons. Pooling all leader-present mechanisms, leaders contribute an average of 9.165 under fixed matching and 7.570 under random rematching, whereas followers contribute 8.028 and 6.552, respectively. After controlling for the mechanism and period and preserving within-group-period dependence, neither pooled role-specific contrast is statistically significant. The adjusted fixed-minus-random difference is 0.690 tokens for leaders (SE=1.249, 95% CI [−1.759,3.138]; Holm-adjusted p=0.581) and 1.578 tokens for followers (SE=1.223, 95% CI [−0.819,3.974]; Holm-adjusted p=0.394). The matching-by-role interaction is also statistically insignificant, $\chi^{2}\left(1\right)=3.033$, p=0.082.

Within DL-RC, the fixed-minus-random difference is positive for both roles. The leader difference is 3.246 tokens (SE=0.963, 95% CI [1.358,5.134]; raw p<0.001; Holm-adjusted p=0.005). The follower difference is 2.587 tokens (SE=1.054, 95% CI [0.520,4.653]; raw p=0.014), but it does not remain statistically significant after correction across the six mechanism-by-role comparisons (Holm-adjusted p=0.071). No role-specific matching contrast is statistically significant in RL or VL-RC. The DL-RC pattern is therefore shared directionally by first and later movers, but the statistical evidence is clearest among first movers.

The supplementary mechanism-by-matching interaction in the full sample is statistically insignificant, $\chi^{2}\left(3\right)=0.649$, p=0.885. Accordingly, the significant conditional DL-RC comparison should not be interpreted as formal evidence that the matching effect is statistically larger in DL-RC than in every other mechanism.

The session-level HC2 analyses produce the same overall direction but wider uncertainty. The overall fixed-minus-random estimate remains 1.394 tokens (SE=1.212, 95% CI [−1.205,3.992], p=0.269). Within leader-present DL-RC, the session-level estimate is 2.931 tokens (SE=0.460, 95% CI [0.954,4.909]; raw p=0.024), but the Holm-adjusted *p*-value is 0.071. The DL-RC leader estimate is also positive in the session-level analysis but does not remain significant after adjustment (Holm-adjusted p=0.076). Because each mechanism-by-matching condition contains only two sessions, these estimates are interpreted as sensitivity evidence.

Taken together, fixed matching does not produce a statistically significant contribution increase across the full sample or across all leader-present or leaderless group-periods. The main conditional pattern is concentrated in DL-RC, where fixed matching is associated with higher contributions and the role-specific difference is most precisely identified among first movers. Stable-group membership may therefore be particularly relevant within the refusal-contingent delegation environment represented by DL-RC, but the experiment does not directly identify reputation, reciprocity, coordination, or accountability as the mechanism producing this pattern.

## 5. Conclusions

We study how leader-selection institutions affect cooperation in a sequential public goods game. Using a unified 2×4 experimental design, we compare a leaderless baseline (control) with three leadership-selection rules (RL, VL-RC, and a novel DL-RC) under both fixed and random matching.

Our analysis yields five main conclusions. First, leadership does not uniformly increase contributions. DL-RC exhibits the lowest observed mean contribution overall, particularly under random rematching; however, the primary omnibus and Holm-adjusted model-based pairwise tests do not establish a general mechanism effect. One session-level sensitivity comparison supports the descriptive ranking. Second, observed contributions differ substantially across the realized acceptance and refusal branches of VL-RC and DL-RC. Because candidates endogenously choose whether to accept leadership, these branch comparisons are conditional and do not identify causal effects of acceptance, refusal, leader absence, or the number of first movers. Third, first movers generally contribute more than later movers, and later-mover contributions are positively associated with observed first-mover contributions. Fourth, the lower observed contribution in DL-RC is descriptively concentrated in the refusal-contingent three-first-mover branch. Finally, fixed matching has a higher observed overall mean than random rematching, but the pooled difference is not statistically significant; the clearest within-mechanism matching contrast occurs in DL-RC.

This study extends prior research (He & Zheng, 2024; Xu et al., 2025) by comparing identical leader-selection mechanisms across fixed-matching and random-rematching environments. The findings reveal an association between the complete DL-RC institutional arrangement and lower observed contributions. In DL-RC, candidate refusal transfers first-mover responsibility to the remaining three group members, and the lower observed contributions are descriptively concentrated in this refusal-contingent branch. This pattern may reflect coordination difficulties, negative inferences from refusal, concerns about the legitimacy of delegated roles, or diminished individual accountability; however, none of these mechanisms were directly measured.

A key limitation of the design is that it does not separately identify the effect of leader multiplicity from the refusal event that triggers delegated leadership. Three delegated first movers emerge only when the initially selected candidate declines the role. Therefore, we interpret the branch comparison as evidence of conditional outcomes within the DL-RC institutional package, rather than as a causal estimate of the effect of multiple leadership. A natural extension would be to assign the first three movers exogenously from the outset, without a prior refusal, thereby isolating leader multiplicity from refusal-contingent delegation.

The experiment is a controlled online laboratory study based on standard VCM parameters and a student sample. Future research could examine whether the observed associations generalize across alternative institutional settings, including heterogeneous endowments, asymmetric information, communication opportunities, and larger groups, as well as to participant populations for whom leadership and collective-action decisions are substantively relevant. Incorporating direct measures of beliefs, expectations, perceived accountability, legitimacy, fairness, and reputational concerns would also help identify the behavioral mechanisms underlying responses to voluntary and delegated leadership.

## Figures and Tables

**Table 1:** Summary of institutional features and hypothesized behavioral implications across leader-selection mechanisms.

	RL	VL-RC	DL-RC
F1. Possibility of multiple leaders	No	No	Yes
F2. Possibility of no leader	No	Yes	No
A1. Respecting individual willingness not to lead	No	Yes	Yes
E1. Free-riding incentive among delegated leaders	No	No	Yes
E2. Strong willingness to lead by the voluntary leader	No	Yes	Yes
E3. No willingness to lead in case of no leader	—	No	—

**Table 2:** Overall average individual contribution by mechanism.

	Control	RL	VL-RC	DL-RC
Contribution	7.052	7.813	7.698	5.578
	(4.988)	(5.961)	(6.170)	(4.794)

Notes: Entries are means of the four individual contributions within a group-period and therefore range from 0 to 20 tokens. Standard deviations across group-period means are in parentheses. Each mechanism contains 160 group-period observations, 64 participants, and four sessions.

**Table 3:** Average individual contribution by mechanism and matching protocol.

	Control	RL	VL-RC	DL-RC
Fixed matching	8.038	7.794	8.053	7.044
	(5.936)	(6.247)	(6.877)	(4.944)
Random rematching	6.066	7.831	7.344	4.113
	(3.587)	(5.700)	(5.392)	(4.179)

Notes: Entries are means of the four individual contributions within a group-period and therefore range from 0 to 20 tokens. Standard deviations across group-period means are in parentheses. Each mechanism-by-matching cell contains 80 group-period observations, 32 participants, and two sessions. Repeated observations from the same participants are accounted for in the mixed-effects analysis.

**Table 4:** Average individual contribution by realized leadership state and matching protocol.

Realized Leadership State	Mean (SD)	*N*	Participants
Panel A. Fixed matching			
Control: no leader	8.037 (5.936)	80	32
RL: one assigned leader	7.794 (6.247)	80	32
VL-RC: accepted, one leader	12.043 (5.782)	47	32
VL-RC: declined, no leader	2.371 (3.469)	33	28
DL-RC: accepted, one leader	10.163 (4.205)	23	28
DL-RC: declined, three leaders	5.785 (4.683)	57	32
Panel B. Random rematching			
Control: no leader	6.066 (3.587)	80	32
RL: one assigned leader	7.831 (5.700)	80	32
VL-RC: accepted, one leader	11.038 (4.555)	39	32
VL-RC: declined, no leader	3.829 (3.425)	41	32
DL-RC: accepted, one leader	7.750 (5.098)	18	30
DL-RC: declined, three leaders	3.056 (3.214)	62	32

Notes: Entries report means of the four individual contributions within a group-period; the contribution measure therefore ranges from 0 to 20 tokens. Standard deviations across group-period means are reported in parentheses. *N* denotes the number of group-period observations, with four individual-period observations in each group-period. Each mechanism-by-matching condition was implemented in two sessions. Participant counts indicate the number of unique participants observed at least once in the corresponding realized state and are not additive across states because the same participant may experience different realized states over the ten periods. The VL-RC and DL-RC states are endogenously generated by candidate acceptance or refusal. Formal conditional comparisons are reported in Appendix B Table A5 and Table A6.

**Table 5:** Individual contributions by mover role and realized leadership state.

Realized Leadership State	Group-Periods	First Mover(s)		Later Mover(s)	
		Mean (SD)	*N* [*P*]	Mean (SD)	*N* [*P*]
Panel A. Fixed matching					
RL: one assigned leader	80	10.300 (7.043)	80 [31]	6.958 (7.227)	240 [32]
VL-RC: accepted, one leader	47	14.191 (5.984)	47 [27]	11.326 (7.034)	141 [31]
DL-RC: accepted, one leader	23	15.130 (5.057)	23 [16]	8.507 (7.041)	69 [27]
DL-RC: declined, three leaders	57	6.450 (6.572)	171 [32]	3.789 (5.453)	57 [25]
Panel B. Random rematching					
RL: one assigned leader	80	11.225 (7.286)	80 [32]	6.700 (6.999)	240 [32]
VL-RC: accepted, one leader	39	16.231 (5.905)	39 [18]	9.308 (7.672)	117 [32]
DL-RC: accepted, one leader	18	13.667 (6.562)	18 [14]	5.778 (7.163)	54 [27]
DL-RC: declined, three leaders	62	3.591 (5.575)	186 [32]	1.452 (3.766)	62 [28]

Notes: Entries report individual-period contributions, ranging from 0 to 20 tokens. Standard deviations are in parentheses. *N* denotes the number of individual-period observations, and *P* in square brackets denotes the number of unique participants observed in the corresponding role and state. In the one-leader states, each group-period contains one first-mover observation and three later-mover observations. In the three-leader state, each group-period contains three first-mover observations and one later-mover observation. Each realized state within a matching protocol is drawn from two experimental sessions. Because the VL-RC and DL-RC states depend on endogenous acceptance or refusal decisions, the table is descriptive. Formal within-group-period comparisons are reported in Appendix B Table A7 and Table A8.

**Table 6:** Inclinations to lead.

		VL-RC	DL-RC
Fixed	Probability of being chosen as a candidate (%)	25	25
		by design	by design
	Conditioning on being a candidate, probability to lead (%)	58.75	28.75
		(0.495)	(0.455)
	Unconditional probability of being a leader (%)	14.69	60.63
		(0.355)	(0.489)
Random	Probability of being chosen as a candidate (%)	25	25
		by design	by design
	Conditioning on being a candidate, probability to lead (%)	48.75	22.50
		(0.503)	(0.420)
	Unconditional probability of being a leader (%)	12.19	63.75
		(0.328)	(0.481)

Notes: The unit of observation is the individual. Standard deviations are in parentheses. In DL-RC, refusal by the selected candidate generates three leaders, which mechanically raises the unconditional probability of being a leader.

**Table 7:** Contributions under fixed matching and random rematching.

Sample	Fixed	Random	$N_{F}/N_{R}$	Fixed − Random	SE	95% CI	*p*-Value
All group-periods	7.732 (6.027)	6.338 (4.983)	320/320	1.394	1.212	[−0.981, 3.768]	0.250
Leader present	8.469 (5.974)	6.965 (5.547)	207/199	1.231	1.183	[−1.087, 3.549]	0.596
Leaderless	6.383 (5.915)	5.308 (3.675)	113/121	1.264	2.013	[−2.682, 5.210]	0.596

Notes: The fixed and random columns report mean group-period contributions, with standard deviations in parentheses. A group-period contribution is the mean individual contribution of the four group members and ranges from 0 to 20 tokens. $N_{F}/N_{R}$ reports the numbers of group-periods under fixed matching and random rematching. The overall estimate is obtained from an individual-period mixed-effects model containing matching protocol and period fixed effects, with random intercepts for the session, participant, and current group-period. The leader-present and leaderless estimates additionally control for the assigned leader-selection mechanism. The overall *p*-value is unadjusted. The two status-specific *p*-values are Holm-adjusted jointly. The overall model contains 2560 individual-period observations from 256 participants, 640 group-periods, and 16 sessions. The leader-present model contains 1624 observations from 192 participants, 406 group-periods, and 12 sessions. The leaderless model contains 936 observations from 124 participants, 234 group-periods, and eight sessions.

**Table 8:** Selected matching-protocol comparisons within realized leadership states and mechanisms.

Realized State	Mechanism	Fixed	Random	$N_{F}/N_{R}$	Fixed − Random	SE	95% CI	Holm *p*-Value
Leader present	RL	7.794 (6.247)	7.831 (5.700)	80/80	−0.038	2.692	[−5.313, 5.238]	1.000
Leader present	VL-RC	12.043 (5.782)	11.038 (4.555)	47/39	0.428	2.800	[−5.060, 5.915]	1.000
Leader present	DL-RC	7.044 (4.944)	4.113 (4.179)	80/80	2.931	0.911	[1.145, 4.717]	0.004
Leaderless	Control	8.038 (5.936)	6.066 (3.587)	80/80	1.972	2.040	[−2.027, 5.971]	0.668
Leaderless	VL-RC	2.371 (3.469)	3.829 (3.425)	33/41	0.583	3.893	[−7.048, 8.213]	0.881

Notes: Descriptive statistics are mean group-period contributions, with standard deviations in parentheses. *N*_F_/*N*_R_ reports group-period counts under fixed matching and random rematching. Model-based estimates are obtained from separate individual-period mixed-effects models containing matching protocol and period fixed effects, with random intercepts for the session, participant, and current group-period. Holm correction is applied across the three-leader-present mechanism comparisons and separately across the two leaderless mechanism comparisons. Leadership status in VL-RC is endogenously determined by the candidate’s decision; the VL-RC comparisons are therefore conditional associations. The adjusted contrast need not equal the unadjusted difference between descriptive means because the realized VL-RC branches occur with different frequencies across periods.

**Table 9:** Role-specific contribution comparisons under fixed matching and random rematching.

Sample	Role	Fixed	Random	$N_{F}/N_{R}$	Fixed − Random	SE	95% CI	Holm *p*-Value
All leader-present groups	Leader	9.165 (7.254)	7.570 (7.803)	321/323	0.690	1.249	[−1.759, 3.138]	0.581
All leader-present groups	Follower	8.028 (7.345)	6.552 (7.228)	507/473	1.578	1.223	[−0.819, 3.974]	0.394
DL-RC	Leader	7.479 (6.991)	4.480 (6.335)	194/204	3.246	0.963	[1.358, 5.134]	0.005
DL-RC	Follower	6.373 (6.771)	3.466 (5.989)	126/116	2.587	1.054	[0.520, 4.653]	0.071

Notes: Descriptive statistics are calculated across individual-period observations, with standard deviations in parentheses. $N_{F}/N_{R}$ reports individual-period observations under fixed matching and random rematching. The pooled estimates are obtained from a joint matching-by-role mixed-effects model that controls for the assigned mechanism and period and contains random intercepts for the session, participant, and current group-period. The two pooled role contrasts form one Holm family. The DL-RC rows belong to a separate exploratory family containing six mechanism-by-role contrasts across RL, VL-RC, and DL-RC. In DL-RC, the leader category includes the voluntary first mover in accepted group-periods and the three delegated first movers in refusal-triggered group-periods.

## Data Availability

The data and analysis code supporting the findings of this study are openly available in Mendeley Data: Chen, Dongsheng; Liu, Jing; Zhang, Wenhao; Zheng, Jie (2026), “Leader-Selection Mechanisms and Matching Protocols in Public Goods Games”, Mendeley Data, V1, doi: 10.17632/jcjtsc8fcg.1.

## Note

1 The z-Tree internal subject identifier was stable and was used by the program to track participants across periods. The identifiers displayed to participants, however, were within-period group identifiers rather than stable personal identifiers. These displayed identifiers were reassigned in each period according to the current-period group composition and random ordering. Therefore, in the fixed-matching condition, participants repeatedly interacted with the same group members but could not identify specific individuals through stable displayed labels. In the random-rematching condition, groups were randomly re-formed in each period; repeated encounters were not explicitly ruled out, although participants could not identify repeated partners through stable displayed IDs.

## Appendix A. Experimental Instructions for the Leadership Treatments (Translated from Chinese)

Thank you for your participation in this experiment! Please read the following instructions carefully. If you have any questions, please feel free to ask us. Please note that you cannot communicate with other participants during the experiment.

You will be paid to complete the experiment according to the instructions. Your payoff for the experiment will be determined by your choices and the choices of other participants. At the end of the experiment the tokens that you have earned will be converted into CNY at the exchange rate of 20 tokens = 1 CNY.

### Appendix A.1. Matching Rules [Fixed]

There are 10 periods in total. At the beginning of the experiment, you will be assigned to a group of four participants. You will remain in the same group throughout all 10 periods and will interact anonymously with the other three participants in your group in each period. This means that the composition of your group will not change during the experiment.

### Appendix A.2. Matching Rules [Random]

There are 10 periods of games in total. At the beginning of each period, you will be randomly allocated to a group of four and carry out the experimental task anonymously with the other three participants in your group for that period. The composition of each group is randomly determined for each period of play. This means that you form a group with different participants in different periods.

### Appendix A.3. Playing Rules

At the beginning of each period, every participant receives 20 tokens. Your task is to decide how you use your endowment. You have to decide how many of the 20 tokens you want to contribute to a project and how many of them to keep for yourself.

Your payoff consists of two parts:

- The tokens which you have kept for yourself (i.e., 20 tokens—your contribution to the project);
- The payoff from the project. This payoff is calculated as follows: The amount of the project equals 1.6 times the tokens invested in the project by all four group members, which is then shared equally among the members of the group (i.e., your payoff from the project = 0.4 × the total contribution of all four group members to the project).

The experiment will take place in two stages chronologically, which are the **First Mover(s) Determination and Allocation Stage** and **Later Mover(s) Allocation Stage**.

#### Appendix A.3.1. First Stage: First Mover(s) Determination and Allocation Stage

**[Specific to the treatment RL]**

At this stage, one of the four group members will be randomly selected as the first mover, and the other three members will automatically become the later movers. The selection procedure is independent in each period.

**[Specific to the treatment VL-RC]**

At this stage, one of the four group members will be randomly selected as the candidate of the first mover. He has to decide whether to play the role of the first mover. If he accepts, the other three members will automatically become the later movers. If he rejects, the whole group will make contribution decisions simultaneously and independently at the second stage. The selection procedure is independent in each period.

**[Specific to the treatment DL-RC]**

At this stage, one of the four group members will be randomly selected as the candidate of the first mover. He has to decide whether to play the role of the first mover. If he accepts, he will become the sole first mover, and the other three group members will automatically become the later movers. If he rejects, the other three group members will automatically become the first movers, and he will become the sole later mover. The selection procedure is independent in each period.

The first mover(s), if any, decide first on their contributions simultaneously, being aware of the specific number of first movers in the group. This amount can take any integer between 0 and 20, and the amount of tokens each first mover has contributed to the project will be made public to the later mover(s) in the subsequent stage. The later mover(s) will wait at this stage for the first mover(s) to decide.

#### Appendix A.3.2. Second Stage: Later Mover(s) Allocation Stage [Common to All the Treatments]

After being informed of the contribution made by the first mover in the first stage, if any, the later movers choose simultaneously the amount of their endowment they contribute to the project, i.e., any integer between 0 and 20, at this stage. After all members have made their decisions, each one in the group is informed about the role of each member (first mover or later mover), the amount of each member’s contribution, the total amount of the project and the payoff of each member for the current period. To keep anonymity, the true identity of every participant is replaced with a randomly drawn identification number.

### Appendix A.4. Payment Rules [Common to All the Treatments]

The payoff you get in each period is the sum of the tokens that you have kept for yourself and the tokens gained from the project. The calculation formula is as follows:

$$\mathrm{Payoff}\;\mathrm{in}\;\mathrm{each}\;\mathrm{period}=\left(20-\mathrm{your}\;\mathrm{contribution}\;\mathrm{to}\;\mathrm{the}\;\mathrm{project}\right)+\frac{1.6\times \mathrm{the}\;\mathrm{total}\;\mathrm{contribution}\;\mathrm{of}\;\mathrm{all}\;4\;\mathrm{group}\;\mathrm{members}}{4}.$$

The accumulated tokens that you have earned across the 10 periods will be converted into CNY at the exchange rate of 20 tokens = 1 CNY. We will take your payoff plus a 5 CNY show-up fee as your final earnings in the experiment. The resulting amount will be paid to you via Wechat at the end of the experiment.

If you have any questions, please contact the experimenter. If there is no problem, we will start the experiment after all participants confirm.

## Appendix B. Supplemental Analysis and Results

The following tables present the effect size, *p*-value, and 95% confidence intervals (CI).

**Table A1:** Mixed-effects models of individual contribution.

	Full Sample	Fixed Matching	Random Rematching
RL	−0.244	−0.244	1.766
	(2.835)	(3.464)	(2.020)
VL-RC	0.016	0.016	1.278
	(2.835)	(3.464)	(2.020)
DL-RC	−0.994	−0.994	−1.953
	(2.835)	(3.464)	(2.020)
Random rematching	−1.972	—	—
	(2.835)		
RL × Random rematching	2.009	—	—
	(4.010)		
VL-RC × Random rematching	1.263	—	—
	(4.010)		
DL-RC × Random rematching	−0.959	—	—
	(4.010)		
Period fixed effects	Yes	Yes	Yes
Session random intercept	Yes	Yes	Yes
Participant random intercept	Yes	Yes	Yes
Stable-group random intercept	No	Yes	No
Current group-period random effect	No	No	Yes
Individual-period observations	2560	1280	1280
Participants	256	128	128
Sessions	16	8	8
Balanced overall mechanism test	$\chi^{2}\left(3\right)=1.58$	—	—
*p*-value	0.665	—	—
Mechanism omnibus test	—	$\chi^{2}\left(3\right)=0.11$	$\chi^{2}\left(3\right)=4.05$
*p*-value	—	0.990	0.256
Mechanism × matching test	$\chi^{2}\left(3\right)=0.65$	—	—
*p*-value	0.885	—	—

Notes: The dependent variable is the individual contribution in each period, ranging from 0 to 20 tokens. Standard errors are in parentheses. Control is the reference mechanism, and fixed matching is the reference protocol in the full-sample model. All models include period fixed effects and are estimated using restricted maximum likelihood. The balanced overall mechanism test evaluates the three mechanism contrasts after assigning equal weight to the fixed- and random-matching protocols. The fixed- and random-matching omnibus tests are obtained from the corresponding protocol-specific models. Under fixed matching, observations are nested within participants, stable groups, and sessions. Under random rematching, crossed random effects account for participants and current group-periods, together with a session random intercept.

**Table A2:** Primary model-based pairwise comparisons of leader-selection mechanisms.

Comparison	Difference	SE	95% CI	Raw *p*-Value	Holm-Adjusted *p*-Value
Panel A. Overall contrasts					
RL − Control	0.761	2.005	[−3.169, 4.691]	0.704	1.000
VL-RC − Control	0.647	2.005	[−3.283, 4.576]	0.747	1.000
DL-RC − Control	−1.473	2.005	[−5.403, 2.456]	0.462	1.000
VL-RC − RL	−0.114	2.005	[−4.044, 3.816]	0.955	1.000
DL-RC − RL	−2.234	2.005	[−6.164, 1.695]	0.265	1.000
DL-RC − VL-RC	−2.120	2.005	[−6.050, 1.809]	0.290	1.000
Panel B. Fixed matching					
RL − Control	−0.244	3.464	[−7.033, 6.546]	0.944	1.000
VL-RC − Control	0.016	3.464	[−6.774, 6.805]	0.996	1.000
DL-RC − Control	−0.994	3.464	[−7.783, 5.796]	0.774	1.000
VL-RC − RL	0.259	3.464	[−6.530, 7.049]	0.940	1.000
DL-RC − RL	−0.750	3.464	[−7.539, 6.039]	0.829	1.000
DL-RC − VL-RC	−1.009	3.464	[−7.799, 5.780]	0.771	1.000
Panel C. Random rematching					
RL − Control	1.766	2.020	[−2.193, 5.724]	0.382	1.000
VL-RC − Control	1.278	2.020	[−2.681, 5.237]	0.527	1.000
DL-RC − Control	−1.953	2.020	[−5.912, 2.006]	0.334	1.000
VL-RC − RL	−0.488	2.020	[−4.446, 3.471]	0.809	1.000
DL-RC − RL	−3.719	2.020	[−7.677, 0.240]	0.066	0.394
DL-RC − VL-RC	−3.231	2.020	[−7.190, 0.727]	0.110	0.548

Notes: Differences are model-based contrasts in individual contribution. Panel A reports contrasts from the full-sample model after assigning equal weight to the two matching protocols. Panel B is based on the fixed-matching model, which includes random intercepts for the session, stable group, and participant. Panel C is based on the random-rematching model, which includes a session random intercept and crossed random effects for the participant and current group-period. All models include period fixed effects. Holm correction is applied separately across the six comparisons in each panel.

**Table A3:** Session-level HC2 sensitivity comparisons for Result 1.

Comparison	Difference	SE	95% CI	Raw *p*-Value	Holm-Adjusted *p*-Value
Panel A. Overall contrasts					
RL − Control	0.761	1.689	[−3.134, 4.655]	0.664	1.000
VL-RC − Control	0.647	2.485	[−5.084, 6.377]	0.801	1.000
DL-RC − Control	−1.473	1.046	[−3.885, 0.938]	0.197	0.983
VL-RC − RL	−0.114	2.635	[−6.191, 5.963]	0.967	1.000
DL-RC − RL	−2.234	1.365	[−5.383, 0.914]	0.140	0.842
DL-RC − VL-RC	−2.120	2.278	[−7.372, 3.132]	0.379	1.000
Panel B. Fixed matching					
RL − Control	−0.244	1.856	[−5.397, 4.910]	0.902	1.000
VL-RC − Control	0.016	4.734	[−13.127, 13.158]	0.998	1.000
DL-RC − Control	−0.994	1.437	[−4.984, 2.997]	0.527	1.000
VL-RC − RL	0.259	4.683	[−12.743, 13.262]	0.958	1.000
DL-RC − RL	−0.750	1.261	[−4.252, 2.752]	0.584	1.000
DL-RC − VL-RC	−1.009	4.534	[−13.596, 11.578]	0.835	1.000
Panel C. Random rematching					
RL − Control	1.766	2.822	[−6.069, 9.601]	0.565	1.000
VL-RC − Control	1.278	1.514	[−2.926, 5.483]	0.446	1.000
DL-RC − Control	−1.953	1.520	[−6.172, 2.266]	0.268	1.000
VL-RC − RL	−0.488	2.419	[−7.203, 6.228]	0.850	1.000
DL-RC − RL	−3.719	2.422	[−10.443, 3.006]	0.199	0.997
DL-RC − VL-RC	−3.231	0.442	[−4.459, −2.003]	0.002	0.011

Notes: The dependent variable is the mean individual contribution within a session. The full sensitivity model uses all 16 session means and includes the mechanism, matching protocol, and their interaction. The mechanism-by-matching interaction is not statistically significant, F(3,8)=0.52, p=0.678. Panels B and C are based on the eight session means within the corresponding matching protocol. HC2 robust standard errors are reported. Holm correction is applied separately across the six comparisons in each panel. Each mechanism-by-matching cell contains only two sessions; these results are therefore interpreted as sensitivity evidence rather than as a replacement for the primary mixed-effects analysis.

**Table A4:** Mixed-effects test of contribution trajectories.

Joint Test	$\chi^{2}$	df	*p*-Value
Mechanism × period and mechanism × matching × period	128.36	54	<0.001

Notes: The dependent variable is the individual contribution in each period, ranging from 0 to 20 tokens. The model includes the mechanism, matching protocol, period, and all corresponding interactions, with random intercepts for the session and participant. The analysis contains 2560 individual-period observations from 256 participants in 16 sessions. The joint test establishes heterogeneity in contribution paths but does not identify a particular pairwise trajectory difference.

**Table A5:** Conditional comparisons across realized leadership states under fixed matching.

Comparison	Difference	SE	95% CI	Raw *p*-Value	Holm-Adjusted *p*-Value
VL-RC: declined − accepted	−6.624	0.689	[−7.975, −5.273]	<0.001	<0.001
DL-RC: three leaders − one leader	−2.274	0.769	[−3.781, −0.767]	0.003	0.003

Notes: The dependent variable is the individual contribution in each period, ranging from 0 to 20 tokens. Each difference is estimated from a separate individual-period mixed-effects model that includes the realized leadership state and period fixed effects, with random intercepts for the session, stable group, and participant. A negative difference indicates a lower contribution in the declined or three-leader branch. Each model contains 320 individual-period observations from 32 participants, eight stable groups, and two sessions. Holm correction is applied jointly across the four conditional comparisons reported in Table A5 and Table A6. Because the realized branches are endogenously selected, the estimates describe conditional differences and should not be interpreted as causal effects of acceptance, refusal, leader absence, or leader multiplicity.

**Table A6:** Conditional comparisons across realized leadership states under random rematching.

Comparison	Difference	SE	95% CI	Raw *p*-Value	Holm-Adjusted *p*-Value
VL-RC: declined − accepted	−6.146	0.883	[−7.876, −4.417]	<0.001	<0.001
DL-RC: three leaders − one leader	−3.976	0.886	[−5.712, −2.240]	<0.001	<0.001

Notes: The dependent variable is the individual contribution in each period, ranging from 0 to 20 tokens. Each difference is estimated from a separate individual-period mixed-effects model that includes the realized leadership state and period fixed effects, with a session random intercept and crossed random effects for the participant and current group-period. A negative difference indicates a lower contribution in the declined or three-leader branch. Each model contains 320 individual-period observations from 32 participants and two sessions. Holm correction is applied jointly across the four conditional comparisons reported in Table A5 and Table A6. Because the realized branches are endogenously selected, the estimates describe conditional differences and should not be interpreted as causal effects of acceptance, refusal, leader absence, or leader multiplicity.

**Table A7:** Within-group-period first-mover–later-mover contribution gaps under fixed matching.

Realized Leadership State	Gap	SE	95% CI	Raw *p*-Value	Holm-Adjusted *p*-Value
RL: one assigned leader	3.347	1.680	[0.055, 6.639]	0.046	0.093
VL-RC: accepted, one leader	3.960	1.759	[0.512, 7.409]	0.024	0.073
DL-RC: accepted, one leader	6.875	1.857	[3.236, 10.513]	<0.001	<0.001
DL-RC: declined, three leaders	2.568	1.709	[−0.783, 5.918]	0.133	0.133

Notes: The gap is defined within each group-period as the mean contribution of the first mover or movers minus the mean contribution of the later mover or movers. A positive value indicates a higher mean contribution among first movers. Estimates are obtained from a group-period mixed-effects model including realized leadership state and period fixed effects, with random intercepts for session and stable group. The analysis contains 207 leader-present group-periods from 24 stable groups and six sessions. Holm correction is applied across the four role-gap comparisons under fixed matching.

**Table A8:** Within-group-period first-mover–later-mover contribution gaps under random rematching.

Realized Leadership State	Gap	SE	95% CI	Raw *p*-Value	Holm-Adjusted *p*-Value
RL: one assigned leader	4.528	0.512	[3.525, 5.531]	<0.001	<0.001
VL-RC: accepted, one leader	6.911	0.742	[5.456, 8.366]	<0.001	<0.001
DL-RC: accepted, one leader	8.057	1.098	[5.905, 10.209]	<0.001	<0.001
DL-RC: declined, three leaders	2.095	0.584	[0.951, 3.239]	<0.001	<0.001

Notes: The gap is defined within each group-period as the mean contribution of the first mover or movers minus the mean contribution of the later mover or movers. A positive value indicates a higher mean contribution among first movers. Estimates are obtained from a group-period mixed-effects model including realized leadership state and period fixed effects, with a session random intercept. The analysis contains 199 leader-present group-periods from six sessions. Holm correction is applied across the four role-gap comparisons under random rematching.

**Table A9:** Comparison of accepted first-mover contributions in VL-RC and DL-RC.

Matching Protocol	DL-RC − VL-RC	SE	95% CI	Raw *p*-Value	Holm-Adjusted *p*-Value
Fixed matching	1.781	1.990	[−2.120, 5.681]	0.371	0.635
Random rematching	−3.099	3.100	[−9.176, 2.977]	0.317	0.635

Notes: The sample is restricted to accepted first movers in the one-leader branches of VL-RC and DL-RC. The dependent variable is the individual contribution of the accepted first mover. The fixed-matching model includes period fixed effects and random intercepts for the session, stable group, and participant. The random-rematching model includes period fixed effects and random intercepts for session and participant. A positive difference indicates a higher accepted first-mover contribution in DL-RC. Holm correction is applied across the two matching-protocol comparisons.

**Table A10:** Later-mover cooperation in the accepted one-leader branches of VL-RC and DL-RC.

Estimate or Comparison	Estimate	SE	95% CI	Raw *p*-Value	Holm-Adjusted *p*-Value
Panel A. Pooled later-mover contribution model					
DL-RC accepted one-leader branch	−3.015	1.048	[−5.495, −0.536]	0.024	—
Random rematching	−2.755	1.299	[−5.827, 0.318]	0.072	—
Observed first-mover contribution	0.521	0.098	[0.289, 0.752]	0.001	—
Panel B. First-mover response slopes					
Fixed matching: VL-RC	0.756	0.096	[0.529, 0.983]	<0.001	—
Fixed matching: DL-RC	0.356	0.063	[0.208, 0.504]	<0.001	—
Fixed matching: DL-RC − VL-RC	−0.400	0.137	[−0.723, −0.077]	0.022	0.044
Random rematching: VL-RC	0.353	0.080	[0.163, 0.544]	0.003	—
Random rematching: DL-RC	0.472	0.051	[0.351, 0.594]	<0.001	—
Random rematching: DL-RC − VL-RC	0.119	0.116	[−0.156, 0.393]	0.340	0.340
Difference in slope differences	0.519	0.127	[0.218, 0.819]	0.005	—

Notes: The sample is restricted to later movers in the accepted one-leader branches of VL-RC and DL-RC. Panel A reports an individual-period regression of later-mover contributions on the DL-RC branch indicator, matching protocol, observed first-mover contribution, and period fixed effects. Panel B adds interactions between the branch indicator, matching protocol, and observed first-mover contribution. Standard errors are clustered by experimental session. The sample contains 381 later-mover observations from 127 group-periods and eight sessions. Holm correction is applied across the two protocol-specific DL-RC–VL-RC slope-difference tests. The difference in slope differences tests whether the mechanism difference in responsiveness varies between the two matching protocols.

**Table A11:** Mixed-effects model of later-mover contributions.

Explanatory Variable	Estimate	SE	95% CI	*p*-Value
Observed mean first-mover contribution	0.526	0.029	[0.470, 0.583]	<0.001
VL-RC: accepted, one leader	0.456	1.326	[−2.144, 3.056]	0.731
DL-RC: accepted, one leader	−1.963	1.372	[−4.651, 0.726]	0.152
DL-RC: declined, three leaders	−0.622	1.365	[−3.297, 2.053]	0.648
Random rematching	−1.476	1.084	[−3.600, 0.649]	0.173
Period fixed effects	Yes			
Session random intercept	Yes			
Participant random intercept	Yes			
Group-period random intercept	Yes			
Later-mover observations	980			
Participants	188			
Group-periods	406			
Sessions	12			

Notes: The dependent variable is each later mover’s individual contribution, ranging from 0 to 20 tokens. The observed mean first-mover contribution is the mean contribution of the first mover or movers in the same group-period. RL and fixed matching are the reference categories. The model includes period fixed effects and random intercepts for the session, participant, and group-period.

**Table A12:** Descriptive incidence of low first-mover contributions in VL-RC and DL-RC.

Matching Protocol and Mechanism	Share ≤5	First-Mover Observations	Participants
Fixed matching: VL-RC	10.64%	47	27
Fixed matching: DL-RC	54.12%	194	32
Random rematching: VL-RC	7.69%	39	18
Random rematching: DL-RC	73.04%	204	32

Notes: A low first-mover contribution is defined as a contribution of no more than 5 of the 20 available tokens. In one-leader group-periods, the table includes the contribution of the single first mover. In the refusal-contingent three-leader DL-RC branch, it includes the individual contributions of all three first movers. The table is descriptive and reflects both the contribution behavior within each realized branch and the frequency with which each branch occurs.

**Table A13:** Mixed-effects estimates of candidate acceptance in VL-RC and DL-RC.

Comparison	VL-RC	DL-RC	DL-RC − VL-RC	SE	95% CI	Raw *p*-Value	Holm-Adjusted *p*-Value
Overall	57.06%	27.11%	−29.95 pp	8.38 pp	[−46.37, −13.53]	<0.001	<0.001
Fixed matching	62.97%	30.53%	−32.44 pp	16.59 pp	[−64.95, 0.07]	0.0505	0.0505
Random rematching	48.28%	25.21%	−23.07 pp	9.49 pp	[−41.66, −4.48]	0.015	0.030
Mechanism × matching interaction			$\chi^{2}\left(1\right)=0.321$		p=0.571		

Notes: The dependent variable equals one when the randomly selected candidate accepts the first-mover role and zero when the candidate declines. The unit of observation is a candidate-period. The full model contains 320 candidate-period observations from 125 unique candidates and eight sessions. It includes the mechanism, matching protocol, their interaction, and period fixed effects, with random intercepts for session and participant. The reported overall probabilities assign equal weight to the two matching protocols. The fixed-matching model contains 160 candidate-periods and includes period fixed effects and random intercepts for session, stable group, and participant. The random-rematching model contains 160 candidate-periods and includes period fixed effects and random intercepts for the session and participant. Differences and standard errors are reported in percentage points (pp). Holm correction is applied jointly to the fixed- and random-rematching comparisons. The overall contrast is a separate summary comparison.

**Table A14:** Session-level HC2 sensitivity analysis of candidate acceptance.

Comparison	DL-RC − VL-RC	SE	95% CI	Raw *p*-Value	Holm-Adjusted *p*-Value
Overall	−28.13 pp	13.51 pp	[−65.62, 9.37]	0.106	0.106
Fixed matching	−30.00 pp	26.52 pp	[−103.62, 43.62]	0.321	0.321
Random rematching	−26.25 pp	5.15 pp	[−40.56, −11.94]	0.007	0.014
Mechanism × matching interaction	F(1,4)=0.019		p=0.896		

Notes: Candidate acceptance is first averaged within each experimental session. The analysis therefore contains eight observations: two mechanisms × two matching protocols × two sessions. The model includes the mechanism, matching protocol, and their interaction and uses HC2 standard errors. Differences and standard errors are reported in percentage points (pp). Holm correction is applied jointly to the fixed- and random-rematching comparisons. The wide intervals reflect the small number of session-level observations and are reported as a sensitivity analysis rather than as the primary specification.

**Table A15:** Analysis units and sample sizes for candidate acceptance and leadership structure.

Matching	Mechanism	Candidate-Periods	Accepted	Unique Candidates	Group-Periods	Participants	Sessions
Fixed	VL-RC	80	47	31	80	32	2
Fixed	DL-RC	80	23	30	80	32	2
Random	VL-RC	80	39	32	80	32	2
Random	DL-RC	80	18	32	80	32	2
Unadjusted Fisher exact tests of equal candidate acceptance rates							
Fixed: VL-RC vs. DL-RC		p<0.001					
Random: VL-RC vs. DL-RC		p<0.001					
Overall: VL-RC vs. DL-RC		p<0.001					

Notes: A candidate-period is one decision by the randomly selected candidate to accept or decline the first-mover role. Because candidates may be selected in more than one period, the number of unique candidates is smaller than the number of candidate-periods. A group-period is one four-person group’s realization in one period. Each mechanism-by-matching cell contains 320 individual-period observations. The Fisher exact tests are unadjusted descriptive comparisons and do not account for repeated candidate decisions; the mixed-effects estimates in Appendix B Table A13 constitute the primary inference.

**Table A16:** Overall and leadership-status-specific matching-protocol comparisons.

Analysis	Sample	Fixed − Random	SE	95% CI	Raw *p*-Value	Holm *p*-Value	Observations	Participants	Sessions
Individual-period	Overall	1.394	1.212	[−0.981, 3.768]	0.250	–	2560	256	16
Individual-period	Overall, mechanism adjusted	1.394	1.257	[−1.070, 3.858]	0.268	–	2560	256	16
Individual-period	Leader present	1.231	1.183	[−1.087, 3.549]	0.298	0.596	1624	192	12
Individual-period	Leaderless	1.264	2.013	[−2.682, 5.210]	0.530	0.596	936	124	8
Session-level HC2	Overall	1.394	1.212	[−1.205, 3.992]	0.269	–	16	–	16
Session-level HC2	Overall, mechanism adjusted	1.394	1.257	[−1.373, 4.161]	0.291	–	16	–	16
Session-level HC2	Leader present	0.921	1.188	[−1.820, 3.662]	0.461	0.921	12	–	12
Session-level HC2	Leaderless	1.274	1.738	[−3.195, 5.742]	0.497	0.921	8	–	8

Notes: Positive estimates indicate higher contributions under fixed matching. Individual-period models include period fixed effects and random intercepts for the session, participant, and current group-period. Status-specific models additionally control for the assigned leader-selection mechanism. Holm correction is applied across the leader-present and leaderless comparisons separately within the individual-period and session-level specifications. The session-level models use HC2 standard errors and are reported as sensitivity analyses.

**Table A17:** Matching-protocol comparisons within realized leadership states and mechanisms.

Analysis	State	Mechanism	Fixed − Random	SE	95% CI	Raw *p*-Value	Holm *p*-Value	Observations	Sessions
Individual-period	Leader present	RL	−0.038	2.692	[−5.313, 5.238]	0.989	1.000	640	4
Individual-period	Leader present	VL-RC	0.428	2.800	[−5.060, 5.915]	0.879	1.000	344	4
Individual-period	Leader present	DL-RC	2.931	0.911	[1.145, 4.717]	0.001	0.004	640	4
Individual-period	Leaderless	Control	1.972	2.040	[−2.027, 5.971]	0.334	0.668	640	4
Individual-period	Leaderless	VL-RC	0.583	3.893	[−7.048, 8.213]	0.881	0.881	296	4
Session-level HC2	Leader present	RL	−0.038	2.692	[−11.619, 11.544]	0.990	1.000	4	4
Session-level HC2	Leader present	VL-RC	−0.131	2.542	[−11.067, 10.805]	0.964	1.000	4	4
Session-level HC2	Leader present	DL-RC	2.931	0.460	[0.954, 4.909]	0.024	0.071	4	4
Session-level HC2	Leaderless	Control	1.972	2.040	[−6.807, 10.751]	0.436	0.872	4	4
Session-level HC2	Leaderless	VL-RC	0.576	3.234	[−13.338, 14.490]	0.875	0.875	4	4

Notes: Positive estimates indicate higher contributions under fixed matching. Individual-period estimates come from separate models containing matching protocol and period fixed effects, with random intercepts for the session, participant, and current group-period. The three-leader-present mechanism comparisons form one Holm family. The two leaderless mechanism comparisons form a separate Holm family. Holm adjustment is applied separately within each specification. VL-RC leadership status is endogenously realized; the VL-RC estimates are conditional comparisons.

**Table A18:** Pooled leader and follower matching-protocol comparisons.

Analysis	Role	Fixed − Random	SE	95% CI	Raw *p*-Value	Holm *p*-Value
Individual-period	Leader	0.690	1.249	[−1.759, 3.138]	0.581	0.581
Individual-period	Follower	1.578	1.223	[−0.819, 3.974]	0.197	0.394
Session-level HC2	Leader	−0.110	1.137	[−2.731, 2.511]	0.925	0.925
Session-level HC2	Follower	1.281	1.320	[−1.762, 4.325]	0.360	0.720

Notes: Positive estimates indicate higher contributions under fixed matching. Leaders and followers are estimated jointly in the individual-period matching-by-role model, which controls for the assigned mechanism and period and contains random intercepts for the session, participant, and current group-period. Holm correction is applied across the two role-specific matching contrasts separately within each specification. The individual-period matching-by-role interaction is $\chi^{2}\left(1\right)=3.033$, p=0.082.

**Table A19:** Role-specific matching-protocol comparisons within leader-present mechanisms.

Analysis	Mechanism	Role	Fixed − Random	SE	95% CI	Raw *p*-Value	Holm *p*-Value	Observations
Individual-period	RL	Leader	−1.409	2.746	[−6.790, 3.973]	0.608	1.000	640
Individual-period	RL	Follower	0.420	2.698	[−4.868, 5.707]	0.876	1.000	640
Individual-period	VL-RC	Leader	−1.384	2.906	[−7.079, 4.312]	0.634	1.000	344
Individual-period	VL-RC	Follower	0.813	2.778	[−4.631, 6.257]	0.770	1.000	344
Individual-period	DL-RC	Leader	3.246	0.963	[1.358, 5.134]	<0.001	0.005	640
Individual-period	DL-RC	Follower	2.587	1.054	[0.520, 4.653]	0.014	0.071	640
Session-level HC2	RL	Leader	−0.925	2.435	[−11.404, 9.554]	0.741	1.000	4
Session-level HC2	RL	Follower	0.258	2.850	[−12.004, 12.521]	0.936	1.000	4
Session-level HC2	VL-RC	Leader	−2.388	1.321	[−8.071, 3.295]	0.212	0.849	4
Session-level HC2	VL-RC	Follower	0.622	3.175	[−13.041, 14.284]	0.863	1.000	4
Session-level HC2	DL-RC	Leader	2.983	0.340	[1.522, 4.444]	0.013	0.076	4
Session-level HC2	DL-RC	Follower	2.964	0.734	[−0.192, 6.121]	0.056	0.281	4

Notes: Positive estimates indicate higher contributions under fixed matching. Each mechanism is estimated using a joint matching-by-role model. Holm correction is applied across all six role-by-mechanism contrasts separately within the individual-period and session-level specifications. The observation counts for the individual-period rows report the complete mechanism-specific model samples rather than role-specific counts. In DL-RC, the leader category combines the voluntary first mover in accepted group-periods and the three delegated first movers in refusal-triggered group-periods.

**Table A20:** Supplementary interaction tests for Result 5.

Model and Hypothesis	Test Statistic	Degrees of Freedom	*p*-Value
Overall mechanism × matching interaction	$\chi^{2}=0.649$	3	0.885
Pooled matching × role interaction	$\chi^{2}=3.033$	1	0.082

Notes: The first test evaluates whether the fixed-minus-random contribution contrast differs across control, RL, VL-RC, and DL-RC. The second test evaluates whether the matching-protocol contrast differs between leaders and followers in leader-present group-periods. These interaction tests are supplementary to the primary overall matching comparison and the prespecified conditional comparison families.
